# Reward function compression facilitates goal-dependent reinforcement learning

**DOI:** 10.1038/s41467-026-78295-1

**Published:** 2026-10-08

**Authors:** Gaia Molinaro, Anne G. E. Collins

**Affiliations:** University of California, Berkeley, Berkeley, CA USA

**Keywords:** Human behaviour, Motivation, Reward

## Abstract

Humans can uniquely assign value to novel, abstract outcomes to support reinforcement learning. However, this flexibility is cognitively costly and reduces learning efficiency. We propose that goal-dependent learning initially relies on capacity-limited working memory. With consistent experience, learners create a compressed reward function — a simplified rule — that transfers to long-term memory for automatic evaluation upon receiving feedback. This automaticity frees working memory resources, thereby boosting learning efficiency. Across six experiments, we demonstrate that learning is impaired by the size of the goal space but improves when this space allows for compression. Additionally, faster reward processing correlates with better learning. Although the algorithmic details remain to be established, computational modeling revealed that individual differences in compression efficiency, proxied by reward processing speed, led to higher choice accuracy. Together, our behavioral results and computational models suggest that efficient goal-directed learning relies on compressing complex goals into stable reward functions.

## Abstract (web summary)

People can learn to achieve arbitrary goals, but this flexibility requires cognitive effort. Here, the authors show that the brain resolves this by progressively compressing complex reward information into simplified rules, freeing up working memory to facilitate reinforcement learning.

## Introduction

Reinforcement learning is a powerful process by which agents learn to make decisions based on experienced rewards and punishments, which has seen broad applications across neuroscience, psychology, and machine learning^1^. The study of animal reinforcement learning (RL) has predominantly focused on two outcome categories: primary rewards, which satisfy basic physiological needs (e.g., food, water), and secondary rewards, which derive their value through learned associations with primary rewards (tokens, money, numeric points^2^). This focus has advanced our understanding of RL behavior and its neural basis in dopaminergic signaling^3^. However, primary and secondary rewards do not adequately capture the complexity of human reward processing^4^, especially in contexts involving abstract goals and deliberate planning^5^. For example, people often find activities rewarding in the absence, if not at the expense, of external incentives^6^. Even when the benefits of engaging in an activity are clear, people can attribute value to its successful completion a priori, without needing to directly associate new outcomes with established rewards. Indeed, humans can flexibly imbue novel outcomes with value and use them to guide learning through a process that recruits subcortical reward circuits^7,8^. Together, these findings suggest that humans use a third class of rewards: flexible, goal-dependent outcomes that derive value from current objectives rather than learned associations^9^.

Goal-dependent reinforcement learning implies the construction of a goal-dependent reward function, i.e., a mapping between an outcome space and scalar rewards. While individuals show remarkable flexibility in creating ad hoc reward functions according to current goals, the underlying process is often inefficient^7^. This is illustrated, for example, by the discrepancy between the high frequency at which people set New Year’s resolutions and the low rate at which they complete them^10^. However, the precise cognitive mechanisms by which humans align their value system to current goals—and which aspects make such alignment hard to maintain – remain uncharted. Identifying the key players involved in goal-dependent learning and their respective weaknesses could inform behavioral strategies that help people achieve their objectives in both personal and educational settings.

Setting up custom reward functions likely involves the interplay of multiple cognitive systems^7^. The existence of interactions between RL and executive functions is now well-established^11,12^. In particular, WM can act as a source of information for RL, affording faster or more efficient learning^13,14^. WM has also been proposed to support the verbalization of critical task components^15,16^ and the representation of states, actions, and rewards over which RL operates^17^. However, much less is known about how exchanges between RL and WM support goal-dependent reward functions, and how they might be limited. Building on existing work^7^, we designed a task where participants had to learn which responses led to desired outcomes, but using abstract images labeled as goal/nongoal as feedback instead of standard numeric points. We replicated our finding that people can indeed imbue abstract, novel stimuli with reinforcing properties, but that learning from goal-dependent outcomes resulted in worse learning performance (^7,8^; Experiment 3 in Supplementary note 1), highlighting the trade-offs between flexible goal-dependent value attribution and learning efficiency. This impairment persisted (albeit substantially smaller) when goal images were kept consistent for the entire duration of the task (^8^, Experiment 4 in Supplementary note 1). However, evidence for the precise reason behind such performance improvements remained inconclusive.

**Fig. 1:**
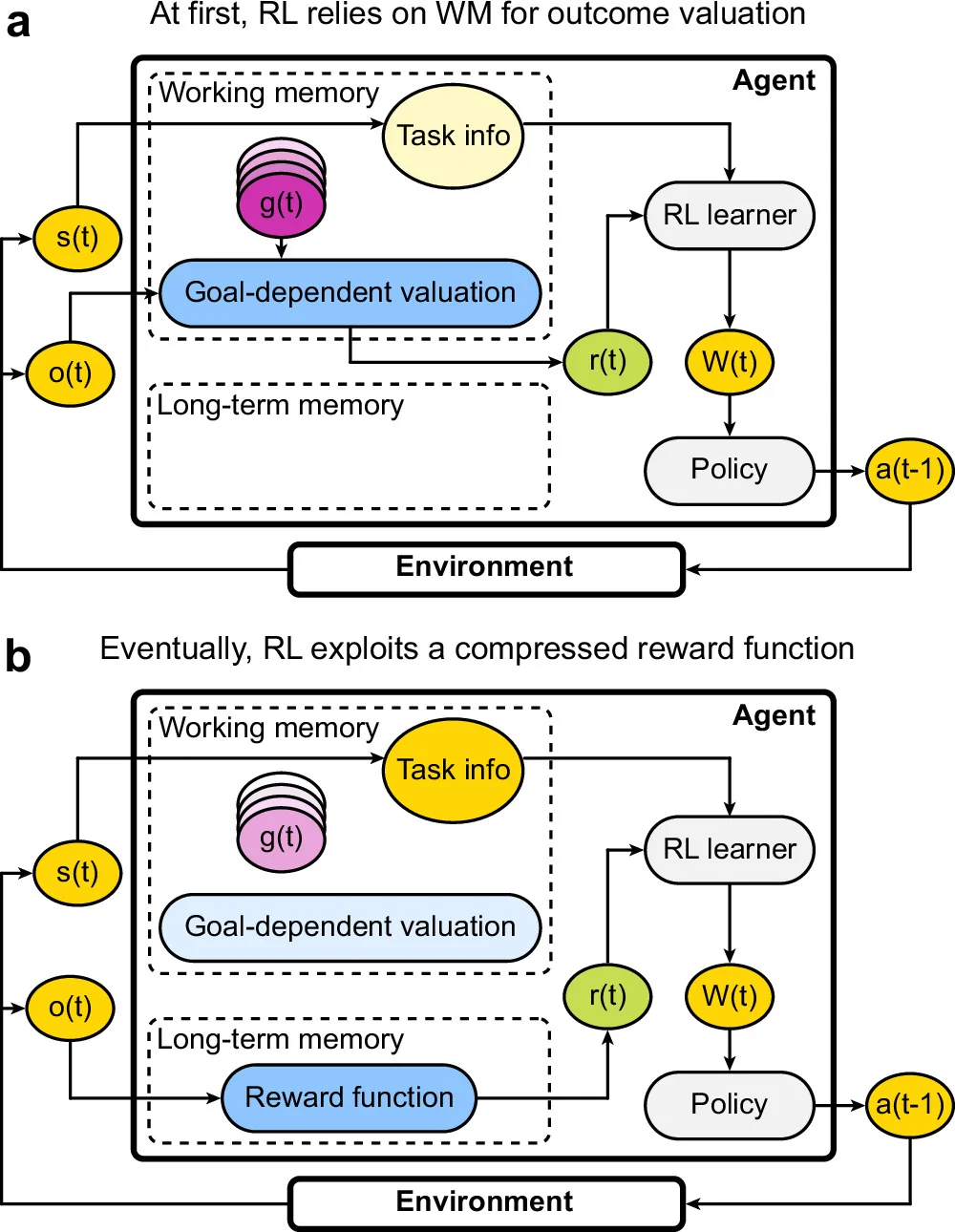
Conceptual model of goal-directed reinforcement learning with reward function compression. In our proposed conceptual model, we break down goal-dependent learning into several parts. Combined with other learning components, an RL learning algorithm informs value estimates *W* on a given trial *t*, which are entered into a policy. The agent selects an action *a* through a joint policy, to which the environment responds by outputting outcome information *o* and a new state *s*. A working memory (WM) component organizes and maintains task-relevant information (here, broadly intended and dubbed the task information), used to support the reinforcement learning (RL) process. **a** Initially, value attribution to *o* is operated by WM, which maintains the current goal (*g*) and supports its matching to the experienced outcome to generate a reward *r* used by the RL agent. WM also holds a trace of recently experienced goals. **b** If goals share enough similar features, a compressed reward function can be created, which replaces active goal maintenance and can, over time, be transferred to long-term memory storage. Eventually, the compressed reward function can be directly employed to assign value to experienced outcomes, sparing resources for other WM tasks.

Here, we propose a dynamic model of goal-dependent reward processing to capture the observed trade-offs between flexibility and learning efficiency. As a real-life example, consider learning chess: a novice might initially hold specific winning positions in mind (queen on d5), then compress these into simple tactical rules (control the center squares), and eventually apply such patterns automatically without conscious effort, freeing mental resources for strategic planning. In our conceptual model, a working memory (WM) component enables us to instantly attribute goal-contingent rewarding properties even to abstract, novel goals. This allows for almost complete freedom in defining what counts as a reward (including usually unrewarding outcomes^18^), which can then be used as a signal for learning (Fig. 1a). However, such freedom comes at a cost, as it requires active maintenance of a goal^19^, using up resources otherwise utilized for the learning process itself^20^. Information stored repeatedly in WM transfers to long-term storage that resists cognitive load^21–24^. Consequently, we propose that pursuing similar goals consistently alleviates the burden on WM through the creation of a compressed reward function from high-dimensional stimuli, which contains the minimum amount of information required to recognize successful outcomes as such while trimming redundant details away (e.g., all goal images are purple). Such a lightweight reward function can eventually be transferred to longer-term memory storage, from where it may easily be retrieved without impinging on other executive functions required to support learning through RL or WM (^11,12,15^; Fig. 1b).

While we remain agnostic with regard to its algorithmic implementation in the brain and formal instantiations of reward function compression itself, our model makes specific predictions, which we test in this study. If WM supports goal evaluation, then learning should worsen as we increase the number of goals to build a reward function across the learning problem (i.e., WM load). In Experiment 1, we manipulate the number of unique goals participants need to learn from, and observe that learning parametrically decays. The model also predicts that performance will benefit from repeated encounters with the same set of goals when it either naturally fits within WM capacity or can be compressed to do so. Only then can the transfer to long-term memory occur, freeing up executive resources to support learning. In Experiment 2, we keep the number of goals constant across blocks, but vary whether the goal space can be compressed along a subset of its dimensions. We find that learning performance significantly improves when people are able to build compressed reward functions, compared to when they are not. In Experiments 3–6, we eliminate alternative mechanisms by which reward functions may be set following instruction, such as cost-free top-down value attribution or cached estimates of reward that are acquired iteratively. While no single experiment eliminates all competing hypotheses at once, the study as a whole tells a coherent story. Taken together and paired with computational modeling, our behavioral findings support reward function compression as a possible mechanism for efficient goal-dependent reinforcement learning.

## Results

### Experimental design

**Fig. 2:**
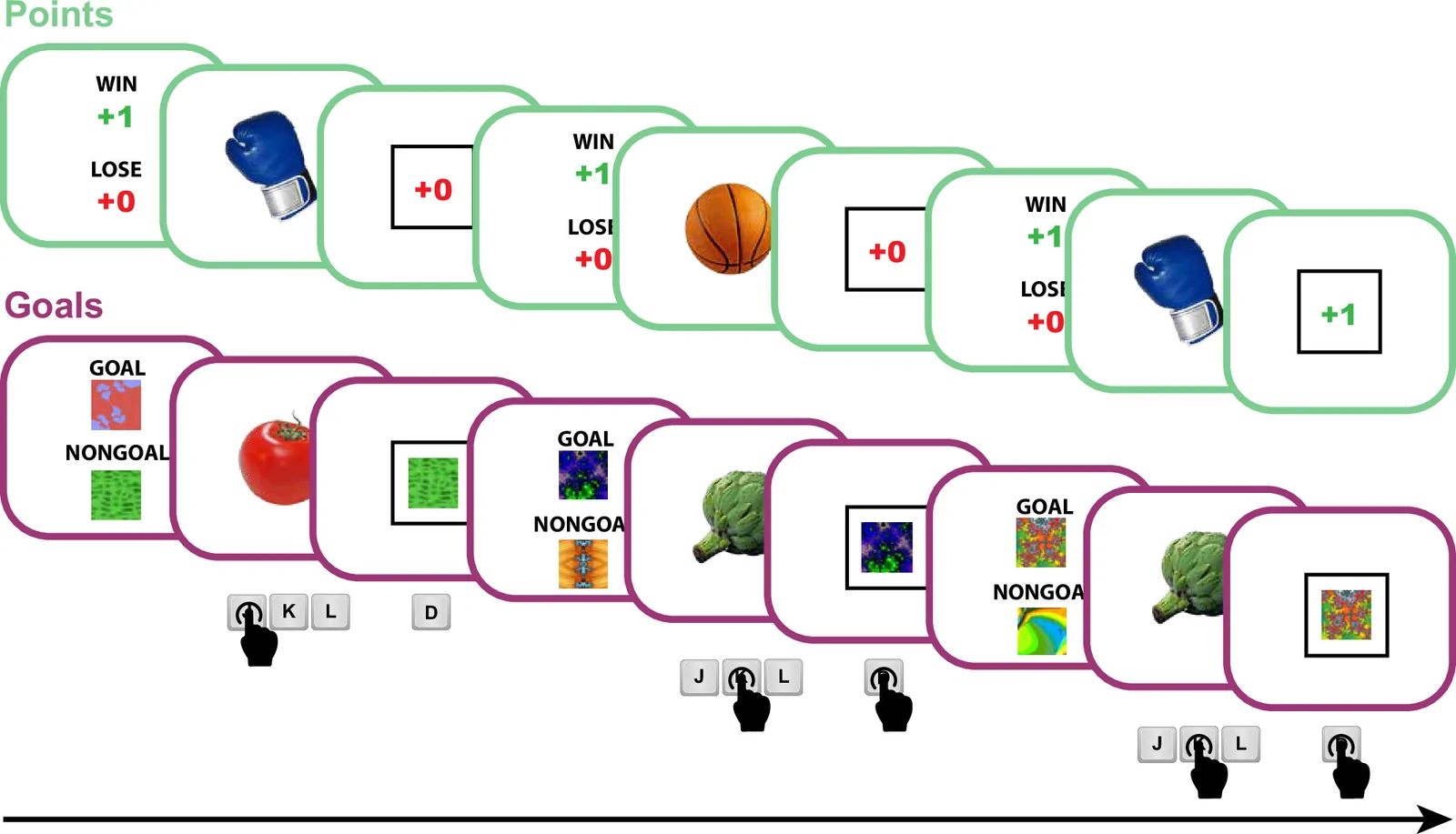
Three example trials for each within-subjects condition. Participants learned the mapping between each of six images and a stimulus-specific, deterministic correct response across several iterations. Within Points blocks, feedback was provided in the form of +1 or +0 messages. Within Goals blocks, goal and nongoal images (independent of stimuli) were used as feedback. Participants were also instructed to collect desirable outcomes by pressing a separate key upon obtaining them while ignoring undesirable outcomes. Actual experiment stimuli were replaced with elements from the Bank of Standardized Stimuli^66^ to preserve image attribution rights.

**Fig. 3:**
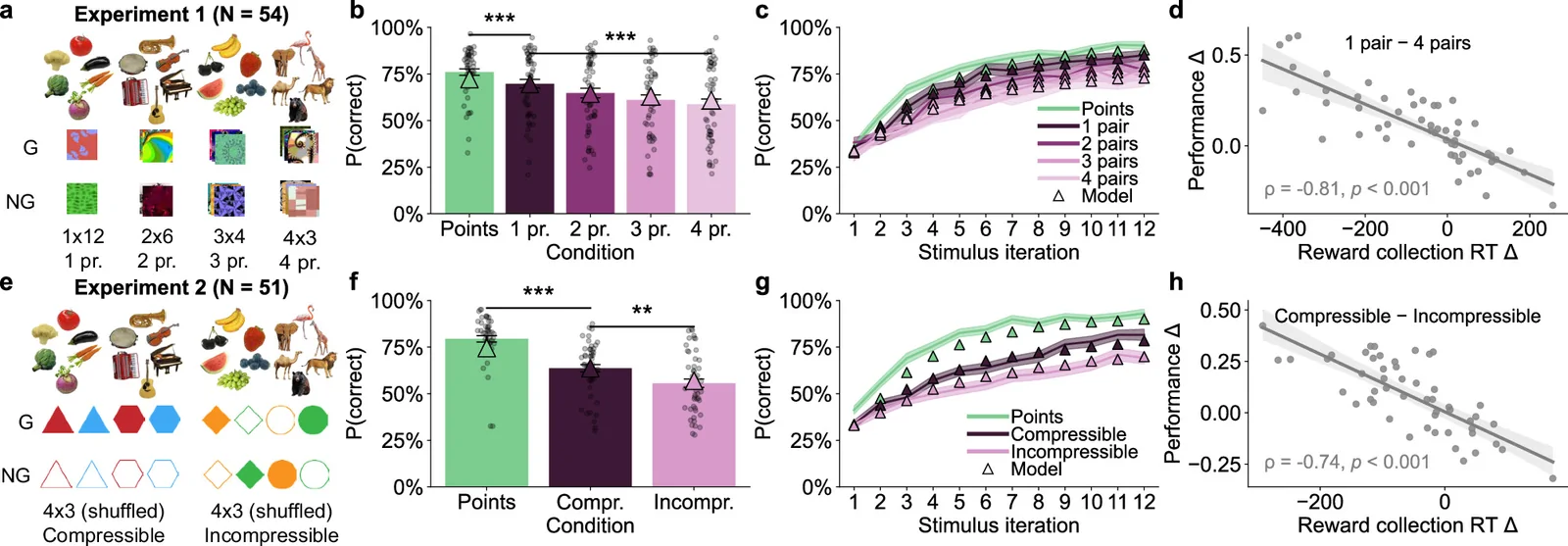
Experiment manipulations, learning performance, and reward collection reaction times (RTs; in ms). **a**, **e** Goal block manipulations. G = Goal image, NG = Nongoal image. **b**, **f** Average choice accuracy across conditions for Experiment 1 (participants *N* = 54) and Experiment 2 (*N* = 51). pr. = pair(s), Compr. = compressible, Incompr. = incompressible. **c**, **g** Learning performance over stimulus iterations. **d**, **h** Correlation between differences in learning performance and reward collection RTs with Spearman’s *ρ* coefficients (both *P* < 0.001). The top row shows results for Experiment 1, the bottom row for Experiment 2. Bars, dots, and solid lines indicate participants' behavior. Triangles indicate simulations from the best-fitting model, where learning rates were informed by reward collection RTs. Error bars and shading show the S.E.M. *** double-sided t-test, *P* < 0.001, ** *P* = 0.002. Actual experiment stimuli were replaced with elements from the Bank of Standardized Stimuli^66^ to preserve image attribution rights.

All experiments share the same fundamental structure, which we adapted from existing tasks^7,13^ where participants had to learn, by trial and error, the correct association between stimuli and specific keys on their computer keyboard (Fig. 2). Both Experiment 1 and Experiment 2 consisted of six blocks, divided into interleaved Points and Goals blocks (two and four of each, respectively) in a pseudorandomized order. Six stimuli were presented in each block and repeated 12 times in an interleaved fashion, for a total of 72 trials. Compared to the original RLWM task^13^, only having blocks with six stimuli in each allowed us to emphasize the RL component relative to value updates directly informed by WM. In the Points blocks, which reflected the structure of a standard RL task and served as a baseline, participants received numeric points as feedback for their responses to the stimuli. Each trial started with a fixation screen (0.5 s), followed by the presentation of +1 and +0 messages indicating desirable and undesirable outcomes, respectively (1.8 s). Participants then saw a stimulus image and were prompted to press one of three keys on their keyboard (1.5 s). Upon responding, they received deterministic feedback showing +1 or +0 messages based on the accuracy of their response (1.5 s) or a warning message if they failed to respond on time. Note that, although feedback was deterministic, participants were in a learning regime for the majority of the task, such that reward prediction errors did not saturate until the very end, if ever (Fig. 3c, g). Participants were instructed to collect desirable outcomes by pressing a separate key within the allotted time while ignoring undesirable outcomes (as in a Go/No-go task). Below, we refer to the latter stage of each trial as reward collection. The Goals blocks followed a similar structure but used abstract fractal-like images instead of numeric points. In each trial, before the stimulus was shown, a pair of fractal images was displayed with the labels Goal and Nongoal to indicate the desired outcome. Fractal images were created using the MandelBrowser and Artmatic Pro applications. Participants needed to encode and recall which images represented goals to learn the correct stimulus-action mappings, then collect goal outcomes on each trial. Correct action selection for each stimulus image deterministically led to the presentation of the current trial’s goal image.

### Experiment 1: Goal-dependent value attribution is impacted by load

Our conceptual model (Fig. 1) proposes that participants will, over time, try to infer a simplified reward function that can be applied to the presented outcome, as opposed to remembering each goal image separately and later comparing it to the experienced outcome. The easier the reward function compression process, the faster goal-dependent reward functions can be ascribed to long-term memory, freeing up cognitive resources for the learning process itself. If only one goal is being pursued across a set of trials, it is easy to compress reward information from the complex fractal goal image to one or a few defining attributes (e.g., color, pattern, etc.) that distinguish it from the nongoal image. When multiple goals are present across trials, such an operation increases the load on WM and thus becomes harder. Therefore, we predicted that modulating the number of goal images in each block would result in performance gains or losses. Returning to our chess example, this would be akin to working toward a single target position across many games vs. juggling a repertoire of several distinct target positions. The latter is harder because resources needed for learning must be spared for active goal maintenance.

In each of the Goals blocks of Experiment 1, outcome images belonged to a set of one, two, three, or four pairs, such that each fractal image pair appeared 12, six, four, or three times per stimulus (Fig. 3a). Block order was pseudorandomized. We additionally ensured that a Points block appeared within the first three and last three blocks. We expected learning performance to be best in the block with a single goal, and worst in the block with four different goals to pursue, with intermediate learning efficiency in the conditions in between. Importantly, conditions did not differ in the amount of information participants had to memorize on a trial-by-trial basis, since only a single goal/non-goal pair was relevant in each trial. Thus, the difference in load was only effective across trials, in keeping with the assumption that participants would consider information from the entire goal space and try to build an overall goal-dependent reward function. Sixty-seven participants completed Experiment 1, 13 of whom did not match our inclusion criteria (see “Methods”). We analyzed data from the remaining 54 participants (74% female, ages 18–25, M = 20.13, SD = 1.48).

Learning performance was higher for Points, compared to Goals trials – even when compared to the easiest, single-goal block (t(53) = 3.74, *P* < 0.001, Cohen’s d = 0.41, 95% CI = [0.03, 0.1]), highlighting the cost of learning from goal-dependent outcomes even in a simple setting and confirming our previous findings^8^. To compare performance across blocks with one, two, three, or four pairs of goal/nongoal outcomes, we ran a linear mixed-effects model predicting average learning performance from the number of outcome image pairs in each Goals block, with random intercepts for participant number. Performance decreased with the number of different outcomes in a block (*β* = 0.073 ± 0.02, *P* < 0.001, 95% CI = [0.04, 0.11]; Fig. 3b, c). By contrast, reward collection *d*’ was not statistically different in Goals (M = 3.86 ± 0.08) and Points trials (M = 3.78 ± 0.09; t(53) = -0.81, *P* = 0.424, d = 0.12, 95% CI = [−0.27, 0.11], BF10 = 0.20 i.e., moderate evidence for the null hypothesis) or across Goals blocks (*β* = −0.01 ± 0.06, *P* = 0.820, 95% CI = [−0.12, 0.10]; Supplementary Fig. 1), showing that the load manipulation did not impact outcome identification and goal maintenance within trials.

In sum, modulating the number of goal/nongoal image pairs from one to four resulted in parametric impairments in learning. This sort of phenomenon, with relatively constrained ceiling-floor effects and proportionally increasing performance in between, is a hallmark of WM^25^, suggesting a similar executive function might be at play in the reward function compression process. However, it is also possible that the observed learning impairments were caused by interference, occurring more frequently in blocks with more unique goal images. To disentangle the two hypotheses, we devised Experiment 2.

### Experiment 2: reward function compression facilitates goal-dependent learning

Our model predicts that the mere repetition of goals is not always sufficient for performance improvements: the goals must be structured in a way that allows for meaningful compression. If participants were simply benefiting from seeing the same goal images repeatedly (as might happen in basic interference accounts), then any repeated goal set should show improvement. However, if our reward function compression hypothesis is correct, performance should improve only when the goal space can be reduced to a simple, memorable rule. Experiment 2 tests this prediction by holding both the number of unique goal images and their frequency of presentation constant while manipulating whether the underlying structure of the goal space permits reward information compression through dimensionality reduction. To achieve this, we replaced fractal images with simpler geometric figures that varied along three different binary axes within each block (Fig. 3e). This resulted in eight figures per block, each having one of two shapes (e.g., square or triangle), colors (e.g., purple or yellow), and fills (full or empty). In Compressible blocks, a single feature was sufficient to separate goal from nongoal images, while other features were irrelevant (e.g., all goal images were full; classification type I in Shepard et al.^26^). In Incompressible blocks, all three features had to be taken into account to determine whether an outcome was a goal or a nongoal image (e.g., goal images were a full orange diamond, a full green circle, an empty green diamond, or an empty orange circle; classification type VI in Shepard et al.^26^). The same rules governed blocks of the same type for a given participant, but rule assignment was pseudorandomized across individuals. Going back to the chess analogy, the two Goal block types are like learning the same number of target positions, but differ in whether all winning positions can be generalized via common features (e.g., controlling the center) or depend on the conjunction of several features, leaving the player to memorize each target board in full. Seventy-one participants completed Experiment 2. After excluding 20 who failed to meet our inclusion criteria, we analyzed data from the remaining 51 participants (80% female, ages 18–32, M = 20.48, SD = 2.27).

As predicted, learning accuracy was significantly higher in Compressible than Incompressible blocks (t(50) = 3.31, *P* = 0.002, d = 0.54, 95% CI = [0.03, 0.13]; Fig. 3f–g). Since the number of occurrences of each goal outcome was identical across the two block types, these results cannot be attributed to differences in goal outcome repetitions (as could have been the case for Experiment 1). Differences in reward collection *d*’ based on the type of Goals block were significant (t(50) = 8.62, *P* < 0.001, d = 1.40, 95% CI = [0.64, 1.03]; Supplementary Fig. 1). However, they were not significantly correlated with corresponding differences in learning performance (*ρ*(49) = 0.17, *P* = 0.226). Moreover, a trial-level logistic mixed-effects model predicting choice accuracy confirmed a significant effect of Goals condition (Compressible vs. Incompressible; *β* = 0.51 ± 0.06, *P* < 0.001, 95% CI = [0.40, 0.63]), even after explicitly controlling for false positive (*β* = −0.40 ± 0.22, *P* = 0.071, 95% CI = [−0.84, 0.03]) and false negative reward collection errors (*β* = −1.58 ± 0.17, *P* < 0.001, 95% CI = [−1.91, −1.26]) on the previous encounter of that stimulus (in addition to stimulus iteration, block number, and their interaction with goal condition). These results suggest that failures in goal identification are unlikely to fully explain the main effect of the Goals condition. Despite new learning, reward collection RTs decreased from the first set of Goals blocks (M = 422.18 ± 13.92) to the second (where goal/nongoal images were reused; M = 316.06 ± 15.90, t(50) = 8.0, *P* < 0.001, d = 0.99, 95% CI = [79.47, 132.77]). This RT decrease was sharper for the Compressible, compared to the Incompressible condition: a linear mixed-effects model predicting reward collection RTs showed a significant interaction between Goals condition type and experiment phase (early vs. late; *β* = −13.58 ± 6.14, *P* = 0.027, 95% CI = [−25.62, −1.53]), controlling for stimulus iteration, block number, and their interaction with the experimental condition. Similar findings applied to learning performance increases across phases (early learning accuracy M = 0.53 ± 0.02, late learning accuracy M = 0.66 ± 0.02; t(50) = −9.45, *P* < 0.001, d = 1.00, 95% CI = [−0.16, −0.10]; Goals condition type by experiment phase interaction *β* = 0.03 ± 0.01, *P* = 0.004, 95% CI = [0.01, 0.04]). Together, these results suggest that learning benefits could not be fully attributed to more habitual choice behavior (Supplementary Fig. 2).

Overall, we find that, although they were never instructed to try to learn mappings between goal outcome features and reward, participants were able to leverage structure in the condition with compressible goal outcomes, which ultimately benefited their learning.

### Reward processing correlates with performance

During the task, participants were instructed to collect positive outcomes (either points or goal images) by pressing a specific key while ignoring negative outcomes, a phase we refer to as reward collection (Fig. 2). In this section, we explore reward collection reaction times (RTs; Supplementary Fig. 1) as a behavioral marker of reward function compression. As participants switch from controlled to more automatic reward processing, their reward collection should become faster. The quicker an individual can recognize and react to an outcome as desirable, the less cognitive effort is presumably expended on this evaluative step. This, in turn, could free up cognitive capacity for the main task of learning new stimulus-response associations. Therefore, we expected individual differences in reward collection RTs across conditions to correlate with corresponding differences in learning performance. By contrast, faster reward collection RTs due to motor habituation, general arousal, or automatic valuation would not systematically differ across the various conditions.

In each experiment, average block-wise individual differences between Points and Goals learning accuracy were strongly correlated with corresponding differences in median log reward collection RTs (Supplementary Fig. 4). Similarly, performance differences between pairs of Goals conditions within the same experiment correlated with corresponding reward collection RT differences (Fig. 3d, h and Supplementary Fig. 5). We sought to ensure that this finding was not driven by differences in engagement or difficulty across conditions. To this end, we reasoned that, if a third, non-specific factor jointly drove block-level changes in choice accuracy and reward collection RTs, we should also expect it to drive choice RTs, and thus a similar correlation between performance and RT. Contrary to this prediction, we found weak or no correlation between choice RT and performance differences across conditions of Experiments 1–2 (Supplementary Figs. 6 and 7). This shows that block-level reward collection RT is uniquely predictive of accuracy. This finding is also counter to the competing hypothesis that goal maintenance impacts choice through a dual-tasking effect, whereby the cognitive load of maintaining the goal to interpret goal-dependent feedback would interfere with and slow down the choice process.

This reliable—albeit correlational—link between reward collection RTs and learning success is difficult to reconcile with simpler alternative models. For instance, an immediate top-down value attribution process would likely be uniformly efficient, leading to consistent reward collection speeds. Similarly, bottom-up explanations that lack an abstraction mechanism would struggle to account for these results—especially in Experiment 2, where goal outcomes were equally frequent but more or less diverse. By contrast, the observed pattern of results strengthens the case for an active reward compression process supporting goal-dependent learning, whereby WM information can be transferred to longer-term storage and be applied without interfering with other executive functions. To test the internal validity of our model, we turn to computational models, leveraging reward collection RTs as a signature of reward function compression.

### Computational models leveraging signatures of reward function compression accurately recover human behavior

To gain a closer understanding of the latent processes guiding learning and decision-making in our task, we fit a set of candidate models that shared a basic RL architecture^27^. Details about computational modeling methods are provided in the “Methods” section. In brief, the value of each stimulus-action pair was updated on each trial through the delta rule^28^. Actions were selected via a noisy softmax policy^29^. This basic RL component had a learning rate *α*, a learning rate multiplier for negative outcomes *ζ*, a fixed inverse temperature *β* = 25, and a free noise parameter *ϵ*. We note that this RL component is meant to capture the reward-dependent learning process, with no claim that it maps onto specific underlying neural mechanisms^1,30^; indeed, we expect that this RL component additionally reflects contributions of working memory to learning^31^. All models also included additional mechanisms, such as forgetting and stickiness, which we found to improve fit. In particular, forgetting proxies RL-WM mixtures that could not be disentangled due to the fixed set-size design of the experiment. An additional learning process tracked stimulus-action associations in an outcome-insensitive, habit-like manner^30^. This component had a separate learning rate *τ* for outcome-insensitive value estimates, and a parameter *κ* that defined the mixture of outcome-sensitive and outcome-insensitive values the RL learner would take into account.

This model, in its base form, best captured the overall learning dynamics, but could not support differences between Points and Goals blocks or within-Goal block sub-conditions. To remedy this, we expanded the base model with separate free parameters for each condition of interest, one mechanism at a time. This resulted in a new family of models where Point trials and each condition of the goal blocks had a specific learning rate, noise parameter, or stickiness bias, etc. Among these, the winning model was the one with separate learning rates *α* for each type of block (Supplementary Fig. 8). In line with previous studies^7^, this result suggests that the value updating process (and not, for instance, the choice selection aspect) was the most negatively impacted by goal-dependent reward attribution.

Treating reward collection RTs as proxies for reward processing efficiency (potentially gained through long-term memory transfer), we also built a family of models where one of the parameters was directly influenced by the block-wise median (log) reward collection RT for the corresponding condition (i.e., Points, or a specific Goals sub-condition; see “Methods” for details) to a degree defined by the parameter *ω*. This model design rests on the assumption that faster RTs indicate more efficient processing and that, if reward-processing gains free up shared resources, they should relate to specific benefits in learning. In contrast, if learning improvements were unrelated to reward processing, linking the two systems would not capture behavior more accurately than a model with parameters independent of reward collection RTs. By applying this method to the learning rate, we were able to capture participants’ behavior (Experiment 1: Fig.3b, c; Experiment 2: Fig. 3f–g) at least as well, and more parsimoniously than the model with free parameters for each experimental condition (Experiment 1: ΔAIC = 0.91, Wilcoxon’s W = 564.0, *P* = 0.124, *r* = 0.21; Experiment 2: ΔAIC = 5.95, W = 225.0, *P* < 0.001, *r* = 0.57; Supplementary Fig. 8). This finding provides formal support for our hypothesis that, when outcome evaluation (proxied by reward collection RT) is efficient, more cognitive resources can be allocated to update stimulus-action associations from feedback. Moreover, the model with learning rates conditioned on reward collection RTs provided a better fit and posterior predictive checks to participants’ data than a model where the choice noise parameter *ϵ* was altered by reward collection RTs (Experiment 1: ΔAIC = 8.24, W = 84.0, *P* < 0.001, *r* = 0.77; Experiment 2: ΔAIC = 8.53, W = 136.0, *P* < 0.001, *r* = 0.69; Supplementary Figs. 9 and 13), showing that the bottleneck in goal-dependent learning occurs at the value-updating stage. We note that these results cannot simply be attributed to a shared variance in the learning process captured by reward collection RT. To illustrate this point, we also fit a model where the learning rate is affected by average choice RT and show that it does not explain behavior as accurately (Experiment 1: ΔAIC = 5.32, W = 302, *P* < 0.001, *r* = 0.51; Experiment 2: ΔAIC = 7.10, W = 238, *P* < 0.001, *r* = 0.56; Supplementary Figs. 9 and 14).

### Additional experiments

We conducted four additional experiments (Supplementary notes 1, 2 and Supplementary Fig. 3) that provide further support to our model by establishing basic phenomena or eliminating some key alternative explanations. Experiments 3 and 4 are re-analyses of previous studies^8^. In Experiment 3, goal images changed on each trial, which made reward function compression impossible and forced participants to attribute value in a one-shot manner. This experiment provides a close replication of McDougle et al.^7^ and shows that goal-dependent learning is more effortful than learning from standard reinforcers. Here, we also find that simple lapses in goal outcome encoding or recognition do not explain impairments in learning performance. This is consistent with the results presented above and suggests that while goal maintenance in itself is efficient, it is a costly process that can utilize resources otherwise allocated to learning. In Experiment 4, goal images were kept consistent across blocks, facilitating reward function compression. We find that, while goal-dependent learning is initially impaired, it eventually reaches similar efficiency levels to learning from standard rewards. We attribute such dynamic changes to a transfer of information from WM to longer-term memory storage. Confirming this hypothesis, we find that individual preferences for goal, over nongoal outcome images—initially neutral—become stronger over time. Although Experiment 2 already showed that repeating goals is not sufficient to achieve performance savings, we provide a more controlled test for this hypothesis in Experiment 5, where we directly manipulate the number of goal outcome repetitions within a block. We find no performance gains for more frequent goal outcomes, which we attribute to the fact that, when the goal space is beyond WM capacity, there is no opportunity for reward function compression, meaning WM is continually burdened. In Experiment 6, we directly address the hypothesis that goal-dependent value attribution is purely done in a one-shot manner, as opposed to being sensitive to the previous history of goal setting. To this end, we manipulate whether goal/nongoal labels remain consistent across trials, controlling for the number of unique goals and how often each is presented, and find that learning is specifically impaired by inconsistent goal/nongoal image labeling across trials. This result suggests that goal-dependent learning takes advantage of repeated goal instances, which we attribute to reward function compression and transfer to longer-term memory storage, and which can make resources available for other online computations.

## Discussion

From mundane activities to long-term ambitions, humans are remarkably capable of setting and pursuing a vast range of goals^9,32^. This flexibility, however, often comes at the cost of efficiency. In this study, we introduced a computational framework that formalizes such trade-offs. Our model posits that goal-dependent learning is initially supported by a fast, but costly, working memory (WM) system that maintains goal representations to evaluate outcomes and inform the reinforcement learning (RL) process^7^. While this allows for one-shot value attribution, it inevitably takes away from the limited capacity of WM, at the cost of either less robust or less precise task-relevant representations, which ultimately impair performance^20^. With consistent experience, however, learners can create a compressed reward function – a simplified representation containing only the essential features needed to identify a goal – which can then be transferred to long-term memory, enabling the reallocation of WM resources and boosting learning efficiency.

Through a series of experiments and computational modeling, we found support for this framework. Experiment 1 demonstrated that goal-dependent learning is parametrically impaired by the number of possible goals: as this number increased within the expected range of WM capacity, learning accuracy decreased. This finding aligns with classic research showing the capacity-limited nature of WM and its impact on concurrent tasks^19,25^, and shows that participants seem to maintain the goal space in WM, despite not needing to do so, since goals were instructed on each trial. This active maintenance is a prerequisite for leveraging executive functions to build a goal-dependent reward function. By keeping the number of goal outcomes constant but varying the complexity of the rule defining a goal, we showed in Experiment 2 that learning significantly improves when the goal space is compressible. This suggests that performance improvements are not merely a function of repeated exposure to the same goals, but rather depend on the ability to extract a simple, generalizable reward function – especially when the goals themselves are too complex to be fully stored in WM. Several mechanisms might support reward function compression, including attention-based feature selection^33,34^. While this specific study allowed for associations between success and nameable rules, similar processes might apply to differently learned reward functions, which may or may not require compression. In addition, the observed correlation between faster reward collection and better learning performance showed that more efficient reward processing interferes less with the resource-demanding process of task pursuit, leading to more efficient learning.

Our findings also rule out competing explanations. In their initial study, McDougle et al.^7^ proposed that the difference in learning rate between Points and Goals could be attributed to occasional lapses occurring in Goals trials, where participants failed to encode or recognize the goal image and subsequently update the probability of repeating the chosen response in future iterations. Excluding this possibility, Experiments 3–4 showed excellent goal outcome recognition in spite of performance differences. In principle, dynamic performance improvements over the task horizon could be explained by cached estimates for the value of an initially neutral outcome (the goal image) that becomes more predictive of reward over time (reminiscent of temporal difference learning; see^35,36^ for similar principles). However, Experiments 5 and 6 showed that manipulating the number of times a goal image is experienced does not, by itself, improve learning—in line with our proposal that reward function compression is necessary for performance gains. Nonetheless, we note that repetition likely fosters long-term reward functions with small enough goal spaces. Another possibility is that goal-dependent reward attribution is done in a completely cost-free, top-down manner that does not take the entire goal spaces into account. By illustrating that performance drops when goal/nongoal labels for the same outcome image are inconsistent with each other, Experiment 6 excludes this possibility. Finally, computational modeling in Experiments 1-2 showed that the performance bottleneck is not at the decision-making step, but rather at the outcome evaluation and integration stages.

Our findings extend a growing body of literature highlighting the interplay between executive functions and RL^11–13,31^. WM in particular is required to support several aspects of RL, including binding relevant elements of the task at hand, retrieving recent episodes, and even replacing RL entirely^11,12,15,17,31^. Here, we focus on the less explored aspect of reward function construction and utilization. We build on prior studies showing that learning from novel, abstract goals is possible but effortful^7,8,37^, and propose a specific mechanism – reward function compression—to explain how this initial cost can be overcome.

Our suggested process of reward function compression and transfer to long-term memory is consistent with long-standing theories, which established that consistent practice allows cognitive processes to shift from controlled, effortful search to automatic, effortless retrieval from memory^21,23,38^. For this to occur, the information maintained in WM must be consistent^22^. A similar dynamic occurs in action-effect learning, where outcomes produced by responses gradually become incorporated into action representations within a few trials^39^, but only under restricted conditions^40^. In our paradigm, it is only when the goal space is stable and simple enough to be held in WM (as in our single goal pair condition of Experiment 1 or the Compressible condition of Experiment 2) that a consistent mapping from outcomes to value can be learned and automatized. However, a formal model of reward functions’ long-term memory transfer remains to be established.

In real life, a major challenge of pursuing arbitrary goals is that they exist in a high-dimensional state space where most features are uninformative^41^. Our work suggests that a key mechanism for efficient learning in such environments is the ability to compress information about rewarding outcomes, ignoring irrelevant features to facilitate generalization and valuation. This resonates with philosophical concepts like value capture, where individuals gravitate towards simple, quantifiable metrics, sometimes at the expense of the richer, more complex values they are meant to represent^42^. Our work suggests compressed reward functions are sought because they help reduce cognitive demands and ensure learning efficiency – albeit towards a different behavior than initially intended.

Our work also opens several further questions. The goals in our experiments were simple and highly simplified. Future research should investigate whether the principles of reward function compression apply in more naturalistic settings, especially with self-imposed goals. Furthermore, the process by which people create compressed reward functions from raw example outcomes—which we did not model in detail—remains to be elucidated. Follow-up work in this direction might benefit from existing literature on function learning. For instance, reward functions may be created based on learned rules, judgments of similarity, or a mixture of the two^43–46^. It would also be interesting to test whether lossy reward function compression leads to systematic errors in value generalization to newly experienced outcomes. More generally, future work should propose and test a model of how reward function compression occurs dynamically on a trial-by-trial basis, and how it is implemented neurally. Moreover, the present study focused on goal-dependent values as a third kind of reinforcer, but goals might also impact primary or secondary rewards^18,47,48^. Systematic differences in reward function compression between deterministic tasks (such as the ones presented here) and probabilistic goal-dependent RL tasks^7^ also remain to be tested. Another avenue for future research would thus be to test the directionality of the association between the performance differences predicted by the reward function compression hypothesis and reward collection RTs, since we can only claim correlation, not causation. For example, faster reward collection RTs could be due to learning improvements, leading participants to expect desirable outcomes more reliably. Finally, our sample was relatively uniform, consisting of university undergraduates. While our exclusion rates were typical for online studies of similar difficulty^18,49,50^, we cannot make conclusions about the applicability of our results to less attentive participants.

In conclusion, our work provides a formal model and empirical evidence for how humans manage the trade-off between flexibility and efficiency in goal-directed learning. We demonstrate that the ability to learn from novel goal-conditioned outcomes is constrained by WM capacity and facilitated by the creation of compressed reward functions that can be transferred to long-term memory. By offering new insights into the cognitive science of motivation, our study provides a potential avenue for developing behavioral and educational strategies to help people achieve their ambitions.

## Methods

All data and analysis code are publicly available at https://doi.org/10.5281/zenodo.22069289^51^.

### Data collection

All participants were recruited through the University of California, Berkeley’s pool of participants (RPP) and received course credit for their participation. Upon providing informed consent, participants completed the experiment online from their own devices. The University of California, Berkeley Institutional Review Board approved the experimental procedure for all experiments (protocol #2024-04-17357). Sex and gender were not considered in the study design. Participants’ sex was determined based on self-report. The experiment was coded using jsPsych (version 6)^52^ and custom software.

To reduce spurious effects due to inattentive participants, exclusion criteria were devised based on visually identified elbow points^53^. Participants were excluded if they: 1) failed to respond on more than 25 trials in total, 2) missed more than 10 trials consecutively, 3) selected the same response more than 15 times in a row, 4) failed to accurately collect rewards in Points trials, as evidenced by either a *d*’ below or equal to 1, or more than 20 reward collection errors, 5) did not understand the instructions for reward collection in Goals trials, as evidenced by a reward collection *d*’ below or equal to 0, or more than 50 reward collection errors in the Goals condition, 6) failed to collect rewards entirely in one or more blocks, 7) had an average response time of less than 300 ms in either the Points or the Goals condition (or both). Participants under one or more such categories were excluded from further analysis. In each new experiment, we aimed to gather data from about 50 participants after applying exclusion criteria, guided by prior literature using similar reinforcement learning and working memory paradigms^7,13^.

### Data analysis

We used the pingouin package (version 0.5.5)^54^ for Wilcoxon signed-rank tests, correlations, Cohen’s d and r effect sizes, and Bayesian Factors, the pymer4 package (0.8.2)^55^ for mixed-effects models, and the scipy package (version 1.16)^56^ for other statistical tests. We used pandas (version 2.3.1)^57^ and numpy (version 2.3.2)^58^ packages for data preprocessing. We used double-sided t-tests to compare average learning performance across pairs of outcome conditions for simplicity, consistency across tests, robustness with sufficiently large sample sizes, and the availability of statistical software. However, results remain unchanged when the non-parametric Wilcoxon signed-rank test is applied to the same comparisons. When comparing average scores across different experiments with unequal variances, we applied Welch’s correction. For null t-test results, we derived Bayes factors via the pingouin package^54^, and interpreted them following Lee and Wagenmakers^59^. All reported correlations are computed as Spearman’s *ρ*. Results for mixed-effects models (MEMs) were estimated via the pymer4 package^60^ and included separate intercepts for each participant. All continuous variables entered in MEMs were first standardized. When block numbers were added to the MEM, they refer to the block number within the corresponding Points or Goals condition. Whenever stimulus iterations were added to the MEM, they were entered as raw values, but we obtained similar results when first taking their log. Categorical variables were first turned into continuous variables, with the following coding scheme. When comparing between Points and Goals, block conditions were coded as 0.5 and −0.5, respectively. When comparing different conditions across Goals blocks, we used 0, −0.5, −1, and −1.5 for one, two, three, and four-pair blocks in Experiment 1 and 0.5 and −0.5 for the Compressible and Incompressible blocks in Experiment 2. When comparing different phases within Experiment 2, we used −0.5 for the first set of blocks and 0.5 for the second. Throughout the manuscript, the ± symbol refers to the S.E.M.

### Computational modeling

All models shared the base mechanisms of a reinforcement learning (RL) agent that tracks *Q* values for each stimulus and response, yielding an *N*_*s*_ × *N*_*a*_ table where *N*_*s*_ = 6 is the number of stimuli, and *N*_*a*_ = 3 is the number of possible responses in each block. Values are initialized at *Q*_0_ = 1/*N*_*a*_ at the beginning of each block, i.e., with uniform probability. Upon receiving feedback *r* on trial *t*, the value of the chosen response (*a*_*c*_) to the presented stimulus (*s*) is updated via the delta rule^28^:

1 $${\delta }_{s,{a}_{c},t}={r}_{s,{a}_{c},t}-{Q}_{t}(s,{a}_{c})$$

2 $${Q}_{t+1}(s,{a}_{c})={Q}_{t}(s,{a}_{c})+\alpha \cdot {\delta }_{s,{a}_{c},t}$$

where *α* is the learning rate. A multiplier *ζ* was applied to the learning rate when outcomes were negative^61^. *Q* values were converted to a policy via the softmax function^29^ with inverse temperature *β* and the noise parameter *ϵ* to capture random errors in the participants’ choices:

3 $${P}_{t}({a}_{c}| s)=(1-\epsilon )\dfrac{\exp (\beta \cdot {Q}_{t}(s,{a}_{c}))}{{\sum }_{i}\exp (\beta \cdot {Q}_{t}(s,{a}_{i}))}+\epsilon \dfrac{1}{{N}_{a}}$$

Because it trades off with other parameters (e.g., the learning rate—potentially undermining parameter recoverability), *β* = 25 was a fixed parameter in all models, as is common practice with similar models and tasks^14,62^. Note that decision noise is captured by the free parameter *ϵ* mentioned above. On each trial, a forgetting rate *ϕ* brought *Q* values closer to 1/*N*_*a*_^7^:

4 $${Q}_{t}={Q}_{t}+\phi ({Q}_{0}-{Q}_{t})$$

After a decision was made and *Q* values were updated, a stickiness value *η* ∈ [−1, 1] was added to *Q*_*t*_(*s*, *a*_*c*_), where *a*_*c*_ is the recently chosen action^63^. This yielded a combined value table *W*, which entered the softmax (Eq. (3)) on the next trial and made the model biased towards (or against) the previous response in a stimulus- and outcome-insensitive fashion. Unchosen actions were left unmodified. Hence:

5 $${W}_{t+1,{a}_{i}}=\left\{\begin{array}{l}{Q}_{t,{a}_{i}}+\eta \,{{\mbox{if}}}\,{a}_{i}={a}_{c}\quad \\ {Q}_{t,{a}_{i}}\,{{\mbox{if}}}\,{a}_{i}\,\ne\, {a}_{c}\quad \hfill\end{array}\right.$$

Stickiness thus captures biases in participants’ choice selection, which drove them to either replicate or avoid the most recently chosen action, irrespective of the outcome or the stimulus being presented.

Models also kept track of chosen stimulus-response pairs in a stimulus-dependent, but outcome-independent fashion^30^. This was implemented through a separate table of values, called *C*, for each stimulus and response. After being initialized at *C* = 0, the value of the chosen stimulus-action pair was updated via the delta rule, but ignoring the outcome:

6 $${C}_{t+1}(s,{a}_{c})={C}_{t}(s,{a}_{c})+\tau \cdot (1-{C}_{t}(s,{a}_{c}))$$

where *τ* is the learning rate. *C* values were mixed with *Q* values as determined by the value of parameter *κ* before entering the softmax (Eq. (3)):

7 $${M}_{t}=(1-\kappa ){Q}_{t}+\kappa {C}_{t}$$

In models with stickiness, *Q*_*t*_ in Eq. (7) was replaced with *W*.

All the parameters listed in the previous sections could optionally vary according to the block’s condition (Points or Goals) and within-block sub-conditions (specific manipulations within Goals blocks). A separate family of models had only one instance of each free parameter, but this value was then scaled by the free parameter *ω* and multiplied by the median log reward collection RT (or choice RT, for alternative models) for the corresponding block and condition. This was intended to model how reward compression efficiency impacted the learning process. We use the median log RTs to mitigate the impact of any shared variance between reward collection/choice RT and trial-by-trial noise.

The fmincon function from MATLAB 2022a (via createOptimProblem and the Python MATLAB engine) was used to obtain the best-fitting parameters for all computational models. The function was used to optimize the log-likelihood of the data under the specified model architecture^64^. All free parameters were bounded between 0 and 1, except for *η* (stickiness parameter), which could take any value between −1 and 1 (since persistence biases are often found, but our deterministic task design could encourage switch behavior) and *ω*, which was constrained between −10 and 10.

Models were evaluated in terms of both predictive and generative validity^65^. We used the Akaike information criterion (AIC) to assess each model’s relative ability to predict the observed data. For differences in model AICs, we report the Wilcoxon signed-rank test statistic. To assess each model’s ability to reproduce the effects of interest, we simulate its behavior with the best-fit 20 times per participant, amounting to roughly 1000 total iterations per model.

We verified that models were identifiable in all key comparisons (Supplementary Fig. 10), and that the winning model’s best-fitting parameters (Supplementary Fig. 11) were recoverable (Supplementary Fig. 12).

### Reporting summary

Further information on research design is available in the Nature Portfolio Reporting Summary linked to this article.

## Supplementary information

Supplementary Information

Reporting Summary

Transparent Peer Review file

## Data Availability

The human data used in this study are available at https://doi.org/10.5281/zenodo.22069289^51^.

## Code availability

All analysis code used in this study is available at https://doi.org/10.5281/zenodo.22069289^51^.
