# Supplementary material for: Reward function compression facilitates goal-dependent reinforcement learning
Source: Nat Commun. 2026 Oct 8;17:11433. doi: 10.1038/s41467-026-78295-1 (PMC13737099; doi:10.1038/s41467-026-78295-1)
Supplement: Supplementary file 1 [file 41467_2026_78295_MOESM1_ESM.pdf]

Supplementary information: "Reward function compression facilitates goal-dependent reinforcement learning"

Gaia Molinaro
University of California, Berkeley

Anne G.E. Collins
University of California, Berkeley

Contents

1 Supplementary note 1 **2**
1.1 Experiment 3 2
1.2 Experiment 4 3
1.3 Experiment 5 4
1.4 Experiment 6 6

2 Supplementary note 2 **8**
2.1 Experiment 1 vs. Experiments 3 and 4 8
2.2 Experiment 2 vs. Experiments 1 and 4 8

3 Supplementary figures **9**

List of figures

1 Reward collection accuracy and RT for Experiments 1 and 2. 9
2 Learning curves and reward collection RTs for Experiment 2's first and last set of blocks. 10
3 Experimental manipulations, learning performance and reward collection accuracy for Experiments 3-6. 11
4 Correlations between differences in performance and differences in reward collection RTs across block types in Experiments 1-6. 12
5 Correlations between differences in performance and differences in reward collection RTs across Goals conditions in Experiments 1-2 and 5-6. 13
6 Correlations between differences in performance and differences in choice RTs across block types in Experiments 1-6. 14
7 Correlations between differences in performance and differences in choice RTs across Goals conditions in Experiments 1-2 and 5-6. 15
8 Model comparison for Experiments 1 and 2 for models with free parameters for separate Goal sub-conditions. 16
9 Model comparison for the main models. 17
10 Model recovery confusion matrix. 18
11 Best-fit model parameters for the winning model. 19
12 Parameter recovery for the best model. 20
13 Model validation with RT-modulated ϵ. 21
14 Model validation with RT-modulated α. 22

1 Supplementary note 1

1.1 Experiment 3

Experiment 3 was previously reported in a separate publication [1] and follows the same structure as the other experiments presented in the current article. Unlike Experiments 1-2, but similar to McDougle et al. [2], goal and nongoal images changed on every trial (Figure 3a). The six blocks of the experiment were equally split among the Points and Goals conditions. Here, we extend previous analyses on the same data set to show that, when reward function compression is prevented (by having goal images change on each trial), learning performance remains inefficient compared to learning from established rewards. Moreover, we show that lapses in goal encoding or recognition that prevent subsequent learning are rare and unlikely to cause the observed differences in performance. To do so, we leverage the "reward collection" stage of our task, which required participants to press a dedicated key upon receiving positive feedback.

One hundred and twenty-five participants completed Experiment 3. Thirty-five participants failed to meet baseline requirements and were excluded from further analysis. Therefore, data from 90 participants (70% female, ages 18-30, M = 20.33, SD = 1.86) were analyzed.

As previously observed in a similar task [2], we found that performance in Goals blocks was above chance (i.e., 33%; M = 0.67 $\pm$ 0.02, μ = 0.33; t(89) = 21.94, $p < 0.001$, d = 2.31, 95% CI = [0.64, 0.7]), meaning participants successfully learned the correct stimulus-response mapping from abstract, novel, neutral images. At the same time, participants' performance in Points blocks (M = 0.8 $\pm$ 0.01) was significantly higher than in Goals blocks (t(89) = 12.65, $p < 0.001$, d = 0.99, 95% CI = [0.11, 0.15]; Figure 3b). When feedback was delivered in the form of numeric points, learning accuracy proceeded faster and reached a higher asymptote (Figure 3c). To compare learning speed in Goals vs. Points blocks, we fit a linear mixed-effects model (MEM) with random intercepts for participant number, predicting average learning performance from condition, stimulus iteration, and within-condition block number. In addition to main effects of condition (β = 0.13 $\pm$ 0.01, $p < 0.001$, 95% CI = [0.12, 0.13]), stimulus iteration (β = 0.14 $\pm$ 0.002, $p < 0.001$, 95% CI = [0.13, 0.14]), and block number (β = 0.04 $\pm$ 0.002, $p < 0.001$, 95% CI = [0.04, 0.05]), we found a small, but significant interaction between block condition and stimulus iteration (β = 0.01 $\pm$ 0.01, p = 0.009, 95% CI = [0.003, 0.02]), with performance improving faster for Points than Goals. Consistent with this, performance was higher for Points (M = 0.93 $\pm$ 0.01) than Goals (M = 0.82 $\pm$ 0.02; t(89) = 7.74, $p < 0.001$, d = 0.74, 95% CI = [0.08, 0.14]) even in the last two stimulus iterations of each block. In addition, we found a significant interaction between condition and block number (β = -0.06 $\pm$ 0.01, $p < 0.001$, 95% CI = [-0.07, -0.06]), with performance benefiting from additional blocks more in the Goals condition than in the Points condition. Nonetheless, differences in performance between Points (M = 0.81 $\pm$ 0.01) and Goals (M = 0.75 $\pm$ 0.02) remained significant even in the last block of each condition (t(89) = 4.33, $p < 0.001$, d = 0.41, 95% CI = [0.03, 0.09]).

Overall, reward collection accuracy (measured as d' – the difference between the Z-scored hit and false alarm rates) was well above chance (i.e., 0; M = 3.62 $\pm$ 0.06; t(89) = 59.75, $p < 0.001$, d = 6.30, 95% CI = [3.5, 3.74]), confirming participants understood the reward collection aspect of the task. While there was a statistically significant difference in reward collection accuracy between Points (M = 3.99 $\pm$ 0.07) and Goals (M = 3.4 $\pm$ 0.07; t(89) = 7.6, $p < 0.001$, d = 0.93, 95% CI = [0.43, 0.74]), even the latter was close to ceiling, with 95% of Goals reward collection responses

being correct (vs. 98% for Points; Figure 3d). If lapses in goal encoding or recognition were the sole culprit for learning inefficiencies in Goals trials, we should observe a correlation between condition-based differences in reward collection accuracy and learning performance. However, this correlation was not statistically significant ($\rho(88) = 0.18$, $p = 0.086$). In previous work [1], we also found that a computational model with subjective rewards based on reward collection decisions (i.e., +1 when participants "collected" and +0 when they did not) did not improve performance relative to a baseline model with objective outcome-based feedback. Therefore, occasional errors in positive outcome recognition are unlikely to explain the observed difference in learning from goal-contingent outcomes relative to familiar rewards.

In sum, Experiment 3 replicates previous findings showing that humans can flexibly imbue neutral outcomes with value, hence leveraging them as learning signals, but that such flexibility comes at a cost [2]. Although plausible, we did not find evidence for a strong contribution of lapses in goal encoding to learning impairments.

1.2 Experiment 4

Experiment 4 was also previously reported [1]. In this version of the experiment, the same two fractals were used as goal and nongoal images throughout the experiment (Figure 3e). Pairs of fractals were created to maximize visual distinctiveness and randomized across participants. Goal and nongoal images were assigned during the instructions, as well as the start of each trial in Goals blocks. As in Experiment 3, participants completed three Points and three Goals blocks, pseudo-randomly interleaved. Keeping a single goal outcome for the whole experiment enabled us to test differences in goal-dependent and point-dependent learning when reward function compression would be easiest to perform. Indeed, in this manipulation, participants only needed to identify a key, distinguishing feature of the goal image to evaluate the outcome (e.g., its dominant color, pattern, etc.). Such information could eventually be transferred into a reward function and stored in long-term memory, making more executive resources available for learning. In addition to performing the main task, participants estimated their liking of goal and nongoal images on Likert scales from 1 ("Not at all") to 5 ("Very much") at three distinct points of the experiment: 1) before any instructions were given on the upcoming task, 2) after participants were informed of which fractal would be used as goal vs. nongoal feedback throughout the experiment, and 3) at the end of the experiment.

One hundred and twenty-one participants completed Experiment 4. The final data set after exclusion comprised 93 participants (73% female, ages 18-27, $M = 20.09$, $SD = 1.82$).

Using a single pair of fractals as goal/nongoal images led to a substantial increase in Goals learning compared to Experiment 3, with the difference between Points and Goals accuracy dropping significantly (from $M = 0.13 \pm 0.01$ to $M = 0.03 \pm 0.01$; $t(181) = 7.24$, $p < 0.001$, $d = 1.07$, 95% CI = [0.07, 0.12]). This drop was specifically driven by the Goals condition (Experiment 3 $M = 0.67 \pm 0.02$, $M = 0.77 \pm 0.01$; $t(181) = -4.99$, $p < 0.001$, $d = 0.74$, 95% CI = [-0.14, -0.06]), whereas Points performance did not significantly differ between the two experiments ($M = 0.8 \pm 0.01$, $M = 0.8 \pm 0.01$; $t(181) = 0.01$, $p = 0.994$, $d = 0.00$, 95% CI = [-0.03, 0.03], $BF10 = 0.16$ suggesting moderate evidence for the null hypothesis).

Although smaller in magnitude compared to Experiment 3, the average difference between Points ($M = 0.8 \pm 0.01$) and Goals learning accuracy ($M = 0.77 \pm 0.01$) remained significant ($t(92) = 3.56$, $p < 0.001$, $d = 0.28$, 95% CI = [0.01, 0.05]; Figure 3f). By contrast, reward collection

accuracies (measured as d') did not statistically differ between the two conditions (Points M = 3.96 ± 0.05, Goals M = 3.93 ± 0.05, t(92) = 0.43, p = 0.667, d = 0.05, 95% CI = [-0.1, 0.15], BF10 = 0.13 moderate evidence for the null hypothesis; Figure 3h) − a result which further discredits the hypothesis that lapses in outcome encoding or recognition are the main cause of learning impairments in the Goals condition. Learning proceeded at different rates within Points compared to Goals blocks (β = -0.01 ± 0.004, p = 0.007, 95% CI = [-0.02, -0.003]; Figure 3g). While performance in Goals trials was initially worse than in Points trials, it eventually reached similar levels, such that choice accuracy in the two conditions was no longer significantly different in the last two stimulus iterations within a block (Points M = 0.93 ± 0.01, Goals M = 0.92 ± 0.01; t(92) = 1.65, p = 0.103, d = 0.12, 95% CI = [-0.0, 0.03], BF10 = 0.42, anecdotal evidence for the null hypothesis). We again found a significant interaction between condition and block number, suggesting stronger meta-learning in Goals than Points trials (β = -0.02 ± 0.004, p < 0.001, 95% CI = [-0.03, -0.01]). Unlike in Experiment 3, we found no significant performance differences between the two conditions in the last block (Points M = 0.82 ± 0.01, Goals M = 0.81 ± 0.01; t(92) = 1.01, p = 0.317, d = 0.09, 95% CI = [-0.01, 0.04], BF10 = 0.19, moderate evidence for the null hypothesis). All MEM effects of condition (β = 0.03 ± 0.004, p < 0.001, 95% CI = [0.02, 0.04]), stimulus iteration (β = 0.14 ± 0.002, p < 0.001, 95% CI = [0.13, 0.15]), and block number (β = 0.03 ± 0.002, p < 0.001, 95% CI = [0.03, 0.04]) were consistent with our findings in Experiment 3. Together, these results suggest that learning from a single outcome, compared to several different instances, benefits goal-conditioned learning.

Supporting the idea that iteratively assigning value to the same goal outcome increases its appetitive value, differences in the subjective liking of goal images, compared to nongoal images, significantly increased from the beginning (M = 0.05 ± 0.15) to the end of the task (M = 0.65 ± 0.18; W = 219.5, p < 0.001, r = 0.78), when they were significantly above 0 (W = 954.5, p = 0.001, r = 0.49).

In sum, goal-dependent learning greatly improved when abstract goal outcomes were fixed, compared to when they were instructed on each trial. This result highlights that imbuing neutral stimuli with value is costly, potentially withdrawing resources from the learning process. With more experience, performance in Goals was not significantly different from that of Points trials. Subjective valuations of goal, relative to nongoal images, also increased throughout the task. These findings are in line with the idea that, given a consistent set of goal features, people can compress the information required to recognize desirable outcomes and spare WM resources to support learning.

1.3 Experiment 5

In principle, the fact that by the end of Experiment 4, participants could perform similarly in the Goals and Points condition could be explained through an iterative process, which facilitates learning from abstract goals with every additional experience. Such a process could, in theory, rely on cached estimates for the value of an initially neutral outcome that becomes more predictive of reward over time (see [3, 4] for similar principles). Experiment 5 directly tested this hypothesis by manipulating the number of times a goal image was repeated for different stimuli in a block. Unbeknownst to participants, the six stimuli in each Goals block were split into three groups, where the same goal/nongoal image pairs were used as feedback 75%, 50%, or 25% of the time, while the rest of the trials involved a new pair of images (Figure 3i). Repeated fractal pairs were unique to each group and shared across stimuli of the same group across blocks, such that participants could not prepare responses during the initial presentation of goal/nongoal outcomes. We additionally

ensured that the correct stimulus-response association was distinct for stimuli of the same group. Repeated fractal pairs were re-utilized for the same group assignment across blocks, whereas novel fractal pairs were unique to each trial in which they were employed. Outcome images and stimulus assignments for each block, condition, and group were pseudo-randomized.

Sixty-seven participants completed Experiment 5, 16 of whom did not meet our inclusion criteria. Data from 51 subjects were analyzed (80% female, ages 18-25, M = 20.68, SD = 1.30).

If goal-contingent value assignment benefited from merely attempting the same goal multiple times, we should expect a significant difference across stimulus groups in the Goals blocks of Experiment 5. To test this, we ran a linear MEM predicting average learning performance in Goals blocks from stimulus groups, with random intercepts for participant number. Contrary to the repetitions hypothesis, we found no statistically significant main effect of stimulus group ($\beta = 0.02 \pm 0.02$, p = 0.355, 95% CI = [-0.02, 0.06]; Figure 3j-k). Specifically, although we found a significant difference between the 75% (M = 0.66 $\pm$ 0.02) and the 50% repetitions groups (M = 0.61 $\pm$ 0.02; t(50) = 2.27, p = 0.027, d = 0.38, 95% CI = [0.01, 0.09]), there were no significant differences between the 75% and the 25% (M = 0.64 $\pm$ 0.02, t(50) = 0.97, p = 0.335, d = 0.14, 95% CI = [-0.02, 0.06], BF10 = 0.24, moderate evidence for the null hypothesis), or the 50% and 25% repetitions groups (t(50) = 1.68, p = 0.1, d = 0.22, 95% CI = [-0.01, 0.07], BF10 = 0.56, anecdotal evidence for the null hypothesis). Similarly, we did not find statistically significant differences across stimulus groups even when only considering the last two iterations of each block (β = -0.001 $\pm$ 0.03, p = 0.967, 95% CI = [-0.06, 0.06]). The lack of a statistically significant difference in learning across groups could not be attributed to ceiling effects, since the overall difference between Points (M = 0.81 $\pm$ 0.01) and Goals performance (M = 0.64 $\pm$ 0.02) remained statistically significant (t(50) = 10.97, $p < 0.001$, d = 1.70, 95% CI = [0.14, 0.2]), even when only considering the last two stimulus iterations of each block (Points M = 0.94 $\pm$ 0.01, Goals M = 0.64 $\pm$ 0.02; t(50) = 19.92, $p < 0.001$, d = 3.32, 95% CI = [0.27, 0.34]). Moreover, overall Goals performance in Experiment 5 was not statistically different from that of Experiment 3 (t(139) = 1.51, p = 0.134, d = 0.26, 95% CI = [-0.01, 0.08], BF10 = 0.52, i.e., anecdotal evidence for the null hypothesis). The same was true when comparing the overall (t(139) = 0.53, p = 0.597, d = 0.09, 95% CI = [-0.04, 0.06], BF10 = 0.21, signifying moderate evidence for the null hypothesis) or late Goals accuracy of Experiment 3 (i.e., in the last two iterations; t(139) = 0.49, p = 0.624, d = 0.09, 95% CI = [-0.05, 0.08], BF10 = 0.21, suggesting moderate evidence for the null hypothesis) to that of the 75% repetitions group of Experiment 5, wherein repetition effects should have been maximal. Learning from standard rewards was also not significantly different between the two experiments (t(139) = -0.4, p = 0.687, d = 0.07, 95% CI = [-0.04, 0.03], BF10 = 0.20 i.e., moderate evidence for the null hypothesis), suggesting the lack of difference in Goals trials was likely not driven by systematic differences between the two samples.

A similar pattern was found for reward collection d', which did not vary statistically across groups ($\beta = 0.13 \pm 0.10$, p = 0.183, 95% CI = [-0.06, 0.32]; Figure 3l), even when considering the last two iterations alone ($\beta = 0.06 \pm 0.08$, p = 0.429, 95% CI = [-0.09, 0.22]). Overall, this set of results is inconsistent with the idea that, by itself, re-experiencing the same goal more times increases its reinforcing power.

Contrary to the proposition that simply re-experiencing the same outcome as a goal image strengthens its reinforcing properties, any effects of repetitions, if present, were too small to be detected in Experiment 5. Instead, learning performance was similar across stimulus groups in the Goals condition.

1.4 Experiment 6

Experiment 6 was designed to test how learning from goal-dependent rewards might be affected by experiencing conflicting instances of an outcome being positive on some occasions and negative on others. To that end, the six stimuli in each block were divided into three groups of two (Figure 3m). In each block, both stimuli of the "pure goal repetitions" group were associated with a single pair of images, each used 12 times per stimulus. This pair of fractals did not differ from block to block. Stimuli of the "conflicting goal repetitions" group were also associated with a single pair of images used 12 times per stimulus. However, fractals belonging to the same pair were presented six times (50% of the time) with a "Goal" label and six times (the remaining 50%) with a "Nongoal" label. To tease apart the beneficial effects of outcome repetition from the detrimental effects of valence conflicts due to label inconsistencies, we also applied a "baseline" condition to two stimuli. Here, fractal image pairs belonged to a set of two, such that each pair was used six times per stimulus (as opposed to the twelve times of the "Pure" goal repetitions group) and always with consistent labels (unlike the "Conflicting" goal repetitions group). Fractal pairs were shared among stimuli of the same group across blocks, and the correct stimulus-response association was distinct for stimuli of the same group. If participants relied on top-down, single-shot value attribution to the current goal outcome, performance for the Conflicting group of stimuli should resemble that of the Pure and Baseline stimuli.

Seventy-one participants completed Experiment 6, 20 of whom did not pass our inclusion criteria. Data from 51 subjects were analyzed (67% female, ages 19-26, M = 20.76, SD = 1.53).

We fit a linear MEM predicting average learning performance in Goals blocks from stimulus group, with random intercepts for participant number. Contrary to the single-shot value attribution hypothesis, we found a significant main effect of stimulus group on learning performance (β = 0.07 ± 0.02, p < 0.001, 95% CI = [0.03, 0.12]; Figure 3n-o). More specifically, there was a significant difference in performance between the Pure (M = 0.62 ± 0.02) and Conflicting (M = 0.55 ± 0.02) Goals stimulus groups (t(50) = 3.34, p = 0.002, d = 0.42, 95% CI = [0.03, 0.12]). There was also a significant difference between the Baseline (M = 0.61 ± 0.02) and Conflicting stimulus groups (t(50) = -2.99, p = 0.004, d = 0.34, 95% CI = [-0.1, -0.02]). By contrast, learning did not statistically differ between Pure and Baseline stimulus groups (t(50) = 0.64, p = 0.522, d = 0.08, 95% CI = [-0.03, 0.06], BF10 = 0.19, moderate evidence for the null hypothesis). Similarly, we found a significant overall effect of stimulus group on performance in the last two stimulus iterations of each block (β = 0.08 ± 0.03, p = 0.007, 95% CI = [0.02, 0.14]). On such trials, performance was higher in the Pure (M = 0.76 ± 0.03) relative to the Conflicting (M = 0.68 ± 0.02) Goals stimulus groups (t(50) = 2.63, p = 0.011, d = 0.33, 95% CI = [0.02, 0.14]), with no statistical difference between the Pure and Baseline (M = 0.71 ± 0.04) outcome conditions (t(50) = 1.61, p = 0.113, d = 0.21, 95% CI = [-0.01, 0.11], BF10 = 0.51, anecdotal evidence for the null hypothesis). However, differences between the Baseline and Conflicting stimulus groups did not reach statistical significance (t(50) = -1.14, p = 0.262, d = 0.12, 95% CI = [-0.08, 0.02], BF10 = 0.28, moderate evidence for the null hypothesis).

Reward collection accuracy, measured as d', also varied by group (β = 0.71 ± 0.11, p < 0.001, 95% CI = [0.50, 0.91]; Figure 3p). Pairwise differences between stimulus groups reflected the results obtained for performance, with significant differences between Pure (M = 3.6 ± 0.08) and Conflicting (M = 2.89 ± 0.1; t(50) = 7.14, p < 0.001, d = 1.09, 95% CI = [0.51, 0.91]) and Baseline (3.48 ± 0.10) and Conflicting stimulus groups (t(50) = -5.49, p < 0.001, d = 0.84, 95% CI = [-0.8,

-0.37]), but not between Pure and Baseline stimulus groups ($t(50) = 1.23$, $p = 0.225$, $d = 0.22$, 95% CI = [-0.08, 0.32], BF10 = 0.31, moderate evidence for the null hypothesis). However, differences in reward collection between Pure and Conflicting stimulus groups were not statistically correlated with the respective difference in learning performance ($\rho(49) = 0.23$, $p = 0.101$), suggesting outcome mislabeling was not a sufficient explanation for the observed differences among groups.

Confirming our results from Experiments 2 and 5, repeatedly experiencing the same outcome as a positive event did not, by itself, improve learning performance if the goal space remains too vast to compress. By contrast, receiving conflicting information about the value of an outcome across different trials had a negative impact on learning. These results do not fit with the hypothesis that attributing rewarding properties to an otherwise neutral stimulus occurs in a purely top-down, one-shot manner. Instead, the observed pattern of results is consistent with the idea that individuals attempt to integrate goal-dependent information across examples of positive outcomes, and utilize limited cognitive resources doing so. Given that only five unique goal images were used in each block of Experiment 6, participants could, in principle, attempt to identify a "compressed" reward function, which would enable them to recognize a desirable outcome as such without having to retrieve its exact perceptual features. However, the "Conflicting labels" condition of Experiment 6 would have actively disrupted participants' ability to apply simplified reward functions across goal outcomes, resulting in performance impairments.

2 Supplementary note 2

In this section, we report additional learning performance comparisons for Experiments 1-2 compared to relevant control Experiments.

2.1 Experiment 1 vs. Experiments 3 and 4

Performance in the four-pair block of Experiment 1 was not statistically different from that of the first Goals block of Experiment 3 [1], where goal/nongoal images changed on every trial (M = 0.57 ± 0.02; t(142) = 0.60, p = 0.549, d = 0.10, 95% CI = [-0.04, 0.08], BF10 = 0.22, signifying moderate evidence for the null hypothesis). By contrast, performance in the one-pair block was not statistically different to that of the first Goals block in Experiment 4 [1], where a single goal/nongoal outcome was employed (M = 0.71 ± 0.02; t(145) = 0.39, p = 0.697, d = 0.07, 95% CI = [-0.06, 0.04], BF10 = 0.20, signifying moderate evidence for the null hypothesis).

2.2 Experiment 2 vs. Experiments 1 and 4

Because of order effects and differences in the types of goal outcomes employed (fractal images vs. simple color shapes), any direct comparisons between Experiment 2 and other experiments are imperfect. Having taken this cautionary note into account, it is remarkable that learning performance in the second block of the Compressible Goals condition (M = 0.71 ± 0.02), when putative compressed reward function for outcome to value mapping would have been established, was not statistically different from that of the first Goals block of Experiment 4 (M = 0.71 ± 0.02; t(142) = 0.22, p = 0.83, d = 0.04, 95% CI = [-0.05, 0.06], BF10 = 0.19, i.e., moderate evidence for the null hypothesis). By contrast, learning performance in the last block of the Incompressible Goals condition (0.61 ± 0.03) remained lower (t(142) = -3.36, p = 0.001, d = 0.58, 95% CI = [-0.16, -0.04]), resembling performance in the four-pair Goals condition of Experiment 1 instead (t(103) = -0.55, p = 0.58, d = 0.11, 95% CI = [-0.06, 0.1], BF10 = 0.24, showing moderate evidence for the null hypothesis). Initially, there was also no statistical difference in learning in the Compressible goals condition (i.e., in the first block; 0.56 ± 0.02) compared to the four unique pairs of goal-nongoal outcomes in Experiment 1 (t(103) = -0.85, p = 0.398, d = 0.17, 95% CI = [-0.1, 0.04], BF10 = 0.28, suggesting moderate evidence for the null hypothesis). However, once a compressed reward function for this condition was established (i.e., in the second block; M = 0.71 ± 0.02), Compressible goals performance significantly improved compared to Experiment 1's four-pair Goals condition (t(103) = 3.44, p < 0.001, d = 0.67, 95% CI = [0.05, 0.2]), with no statistical difference from that of Experiment 1's one-pair Goals condition instead (t(103) = 0.49, p = 0.628, d = 0.09, 95% CI = [-0.05, 0.08], BF10 = 0.23, i.e., moderate evidence for the null hypothesis).

3 Supplementary figures

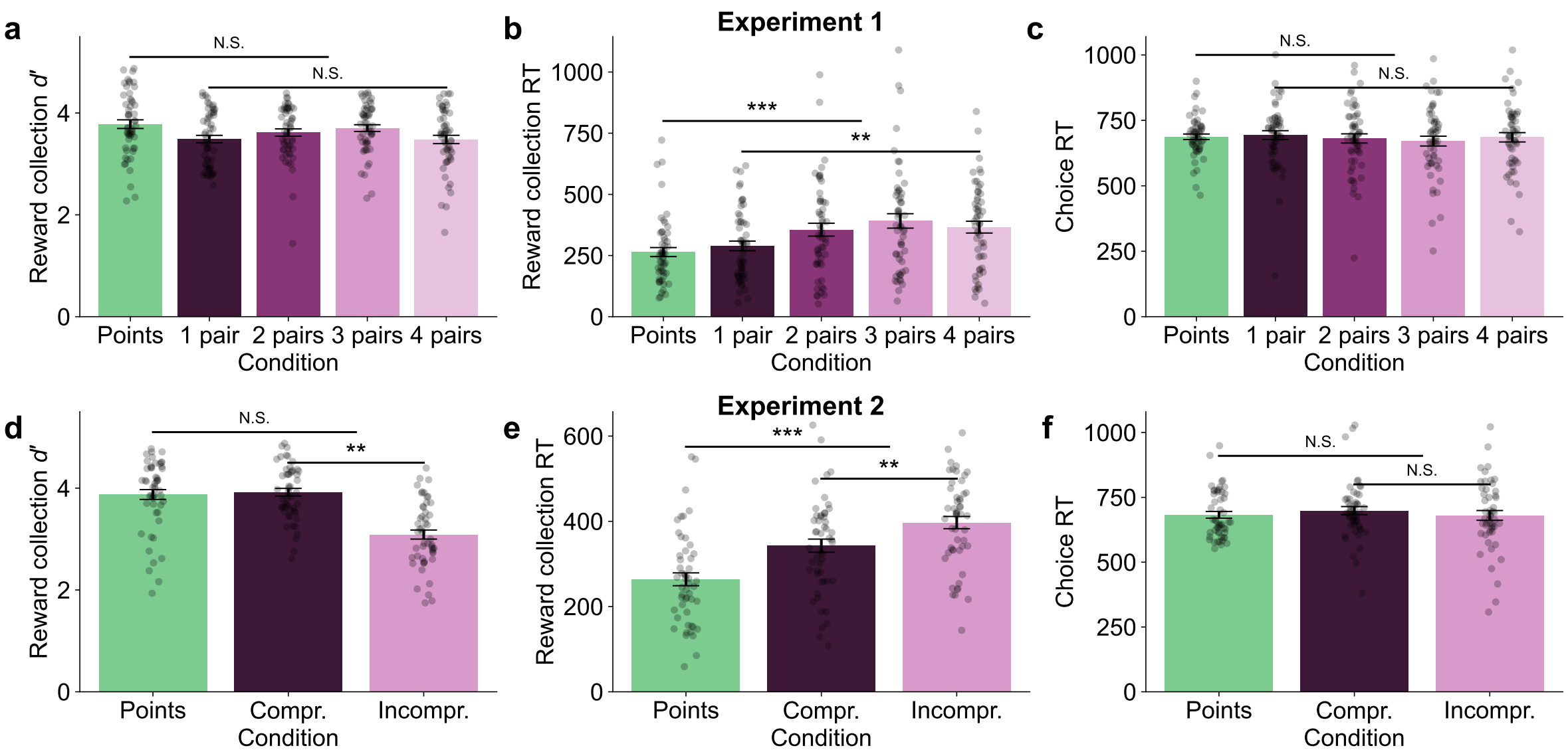

Supplementary Figure 1: **Reward collection accuracy and reaction time (RT; in ms) for Experiments 1 (N = 54) and 2 (N = 51). a, d** Reward collection accuracies across conditions. **b, e** Reward collection RTs in ms across conditions. **c, f** Choice RTs across conditions. Bars indicate participants' average behavior, dots show individual participants' scores. Error bars indicate the S.E.M. Double-sided t-test, *** = $p < 0.001$, ** = $p < 0.01$.

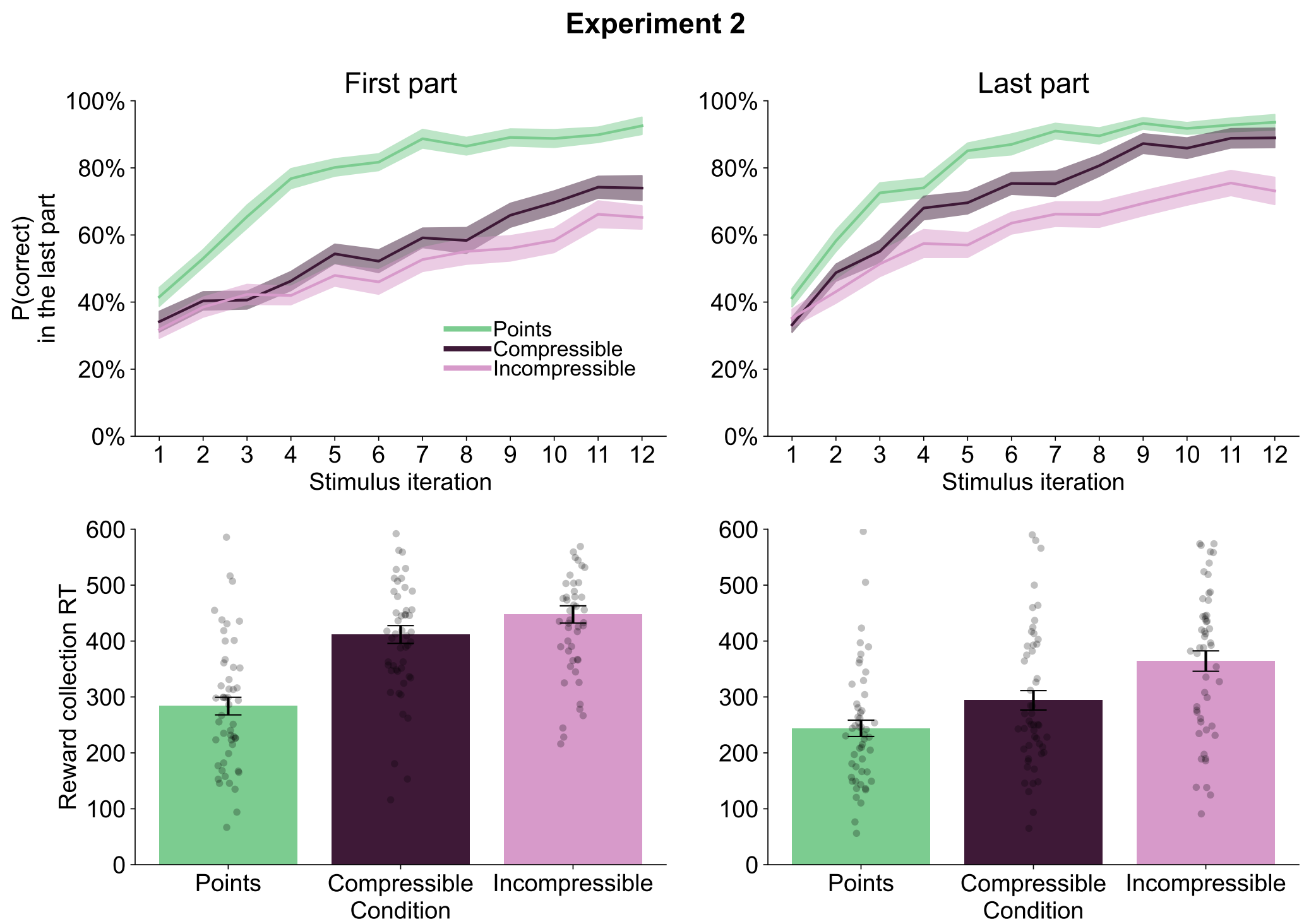

Supplementary Figure 2: **Learning curves and reward collection RTs for Experiment 2's (N = 51) first and last set of blocks.** Bars indicate participants' average behavior, dots show individual participants' scores. Error bars indicate the S.E.M.

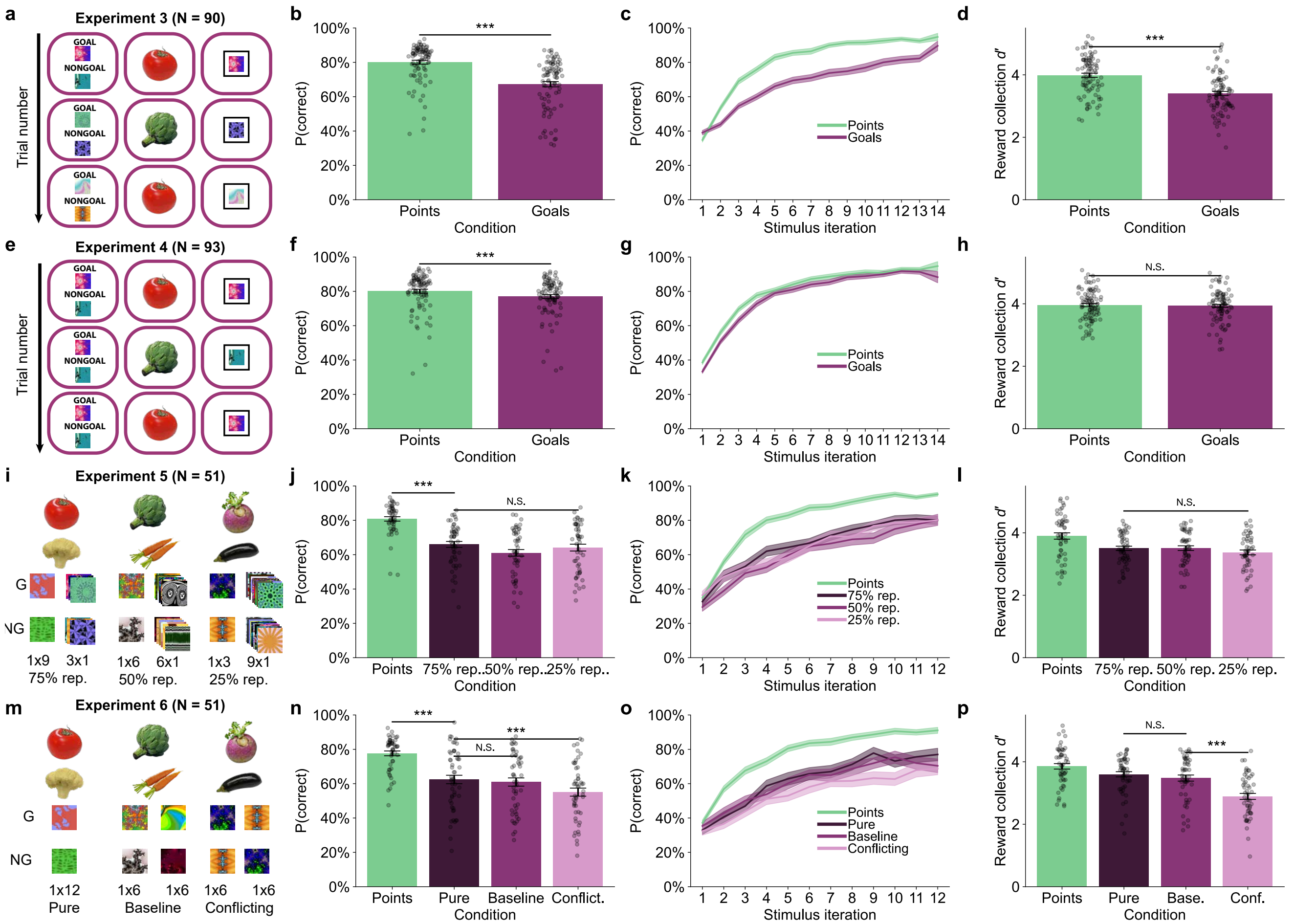

Supplementary Figure 3: **Experimental manipulations, learning performance and reward collection accuracy for Experiments 3 (N = 90), 4 (N = 93), 5 (N = 51), and 6 (N = 51). a, e, i, m** Experimental manipulations for Experiments 3-6. G = Goal image, NG = Nongoal image. **b, f, j, n** Average learning performance across conditions. **c, g, k, o** Learning performance over stimulus iterations. **d, h, l, p** Average reward collection accuracy (d') across conditions. Bars, dots, and solid lines indicate participants' average behavior. Error bars and shading indicate the S.E.M. Double-sided t-test, *** = $p < 0.001$, ** = $p < 0.01$. Actual experiment stimuli were replaced with elements from the Bank of Standardized Stimuli [5] to preserve image attribution rights.

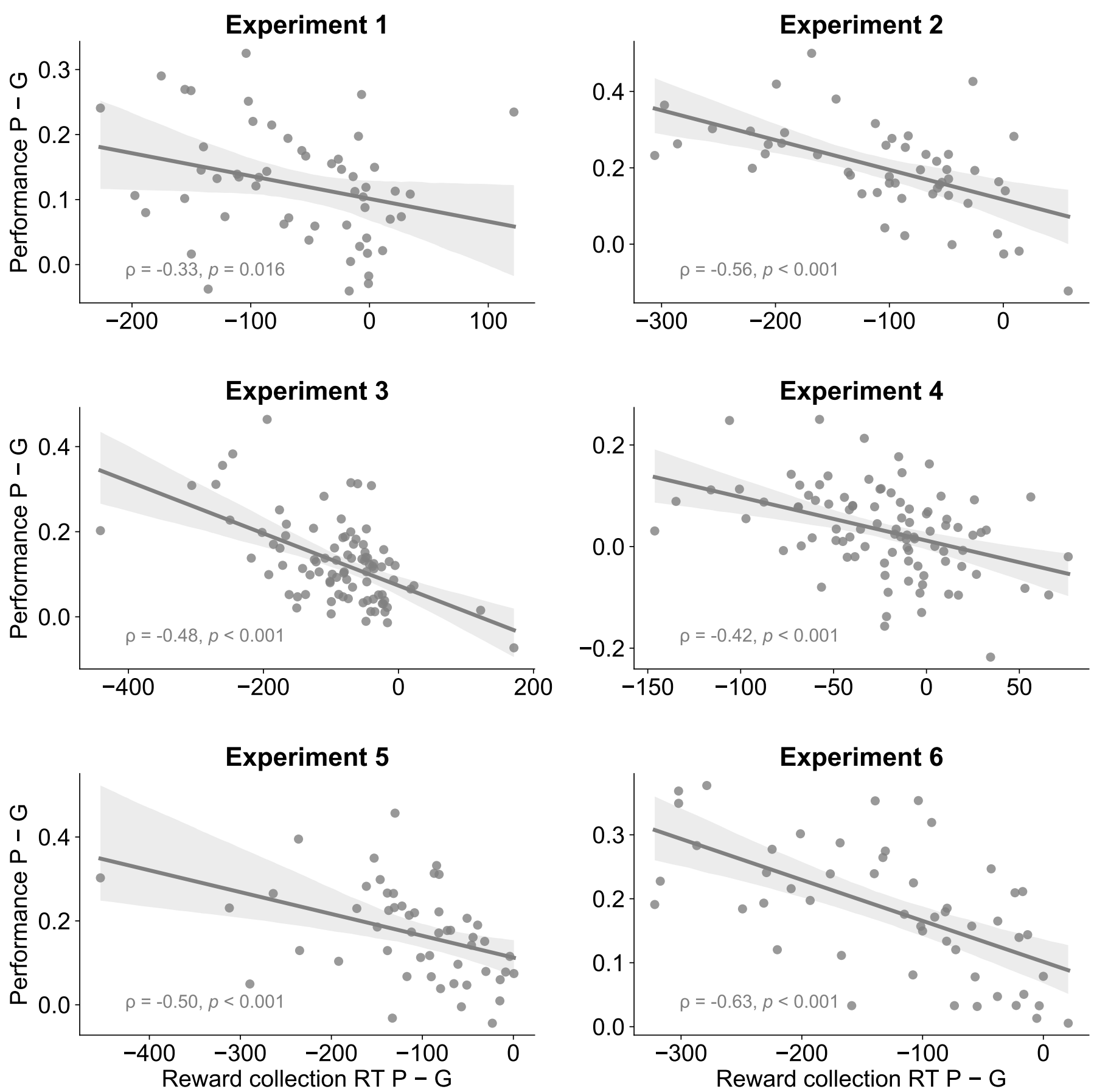

Supplementary Figure 4: **Correlations between differences in performance and differences in reward collection reaction times (RTs; in ms) across block types (Points vs. Goals) in Experiments 1 (N = 54), 2 (N = 51), 3 (N = 90), 4 (N = 93), 5 (N = 51), and 6 (N = 51).** Dots represent individual participants. Shading represents 95% confidence intervals. Spearman's ρ correlation coefficients and p-values are shown on each respective plot.

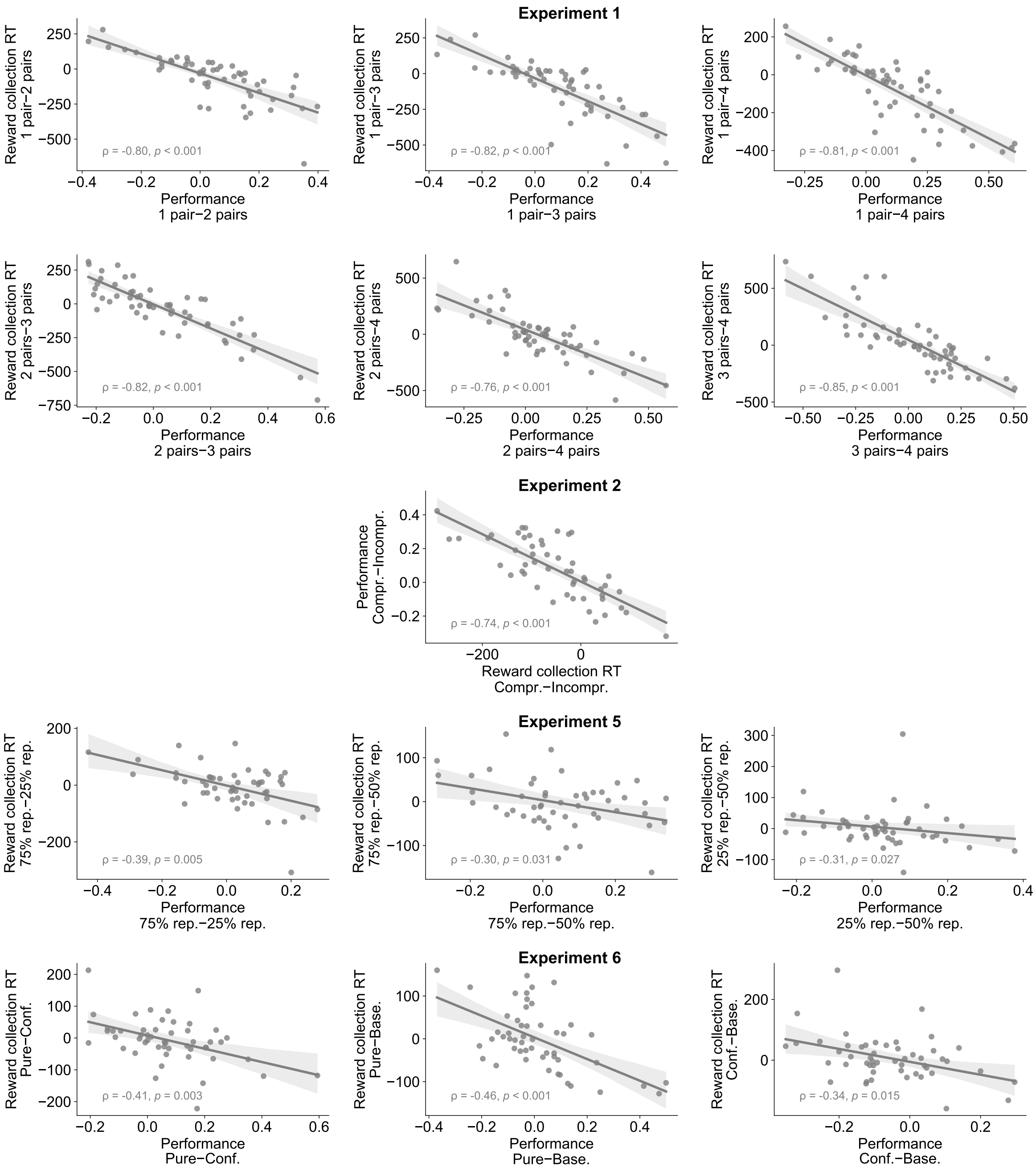

Supplementary Figure 5: **Correlations between differences in performance and differences in reward collection reaction times (RTs; in ms) across conditions of Goals blocks in Experiments 1 (N = 54), 2 (N = 51), 5 (N = 51), and 6 (N = 51).** Dots represent individual participants. Shading represents 95% confidence intervals. Spearman's ρ correlation coefficients and p-values are shown on each respective plot.

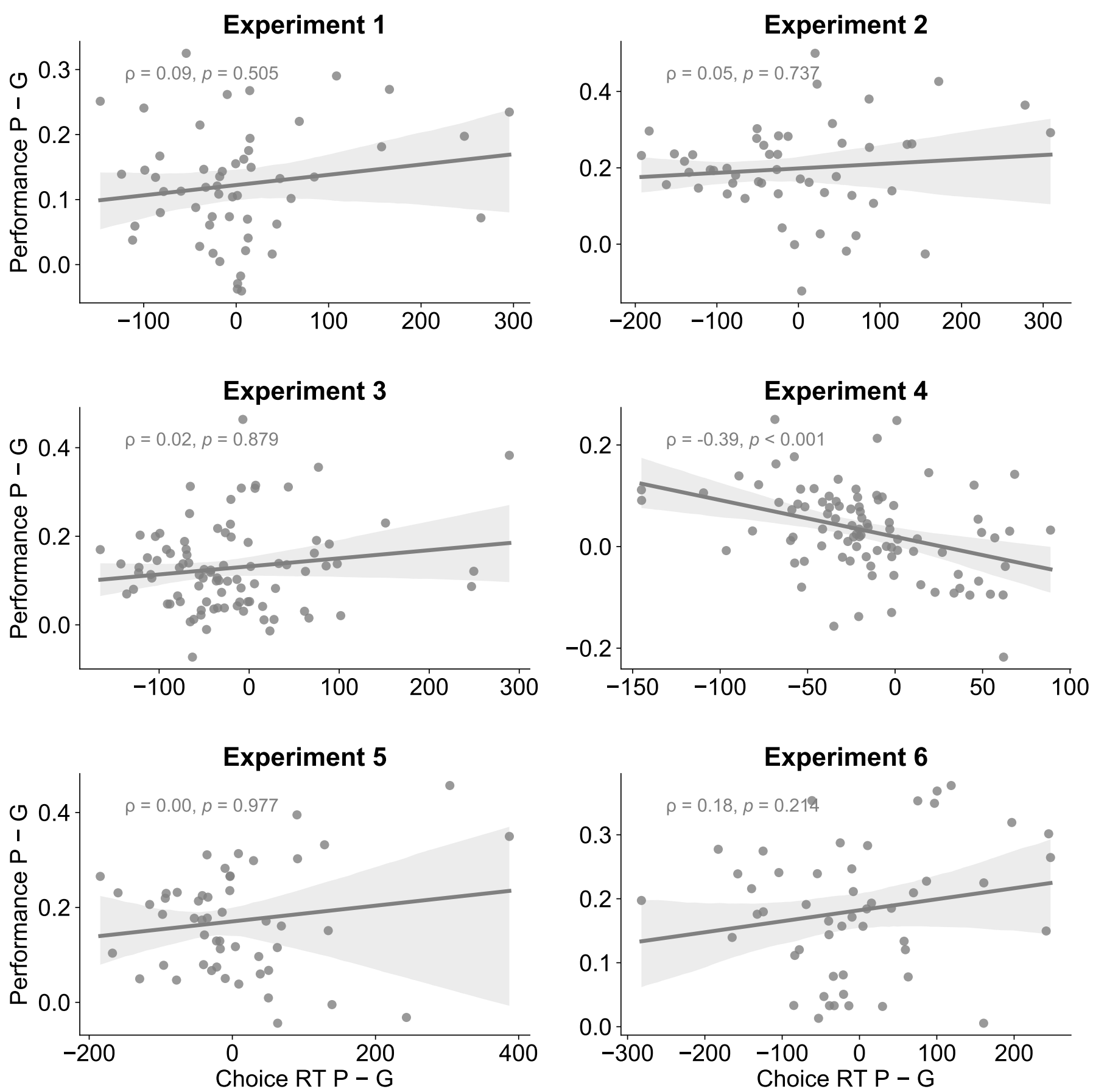

Supplementary Figure 6: **Correlations between differences in performance and differences in choice reaction times (RTs; in ms) across block types (Points vs. Goals) in Experiments 1 (N = 54), 2 (N = 51), 3 (N = 90), 4 (N = 93), 5 (N = 51), and 6 (N = 51)**. Dots represent individual participants. Shading represents 95% confidence intervals. Spearman's ρ correlation coefficients and p-values are shown on each respective plot.

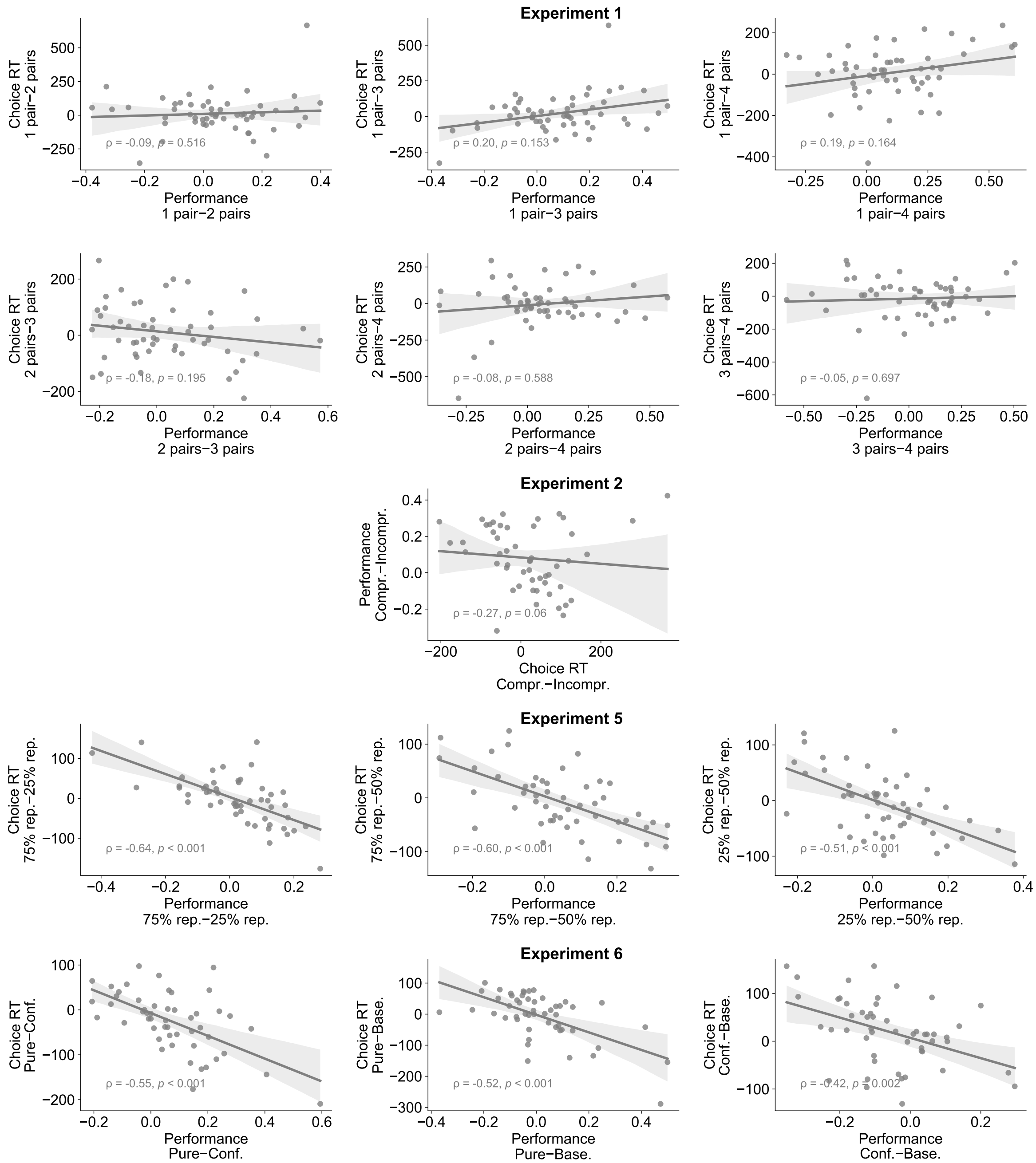

Supplementary Figure 7: **Correlations between differences in performance and differences in choice reaction times (RTs) across conditions of Goals blocks in Experiments 1 (N = 54), 2 (N = 51), 5 (N = 51), and 6 (N = 51).** Dots represent individual participants. Shading represents 95% confidence intervals. Spearman's ρ correlation coefficients and p-values are shown on each respective plot.

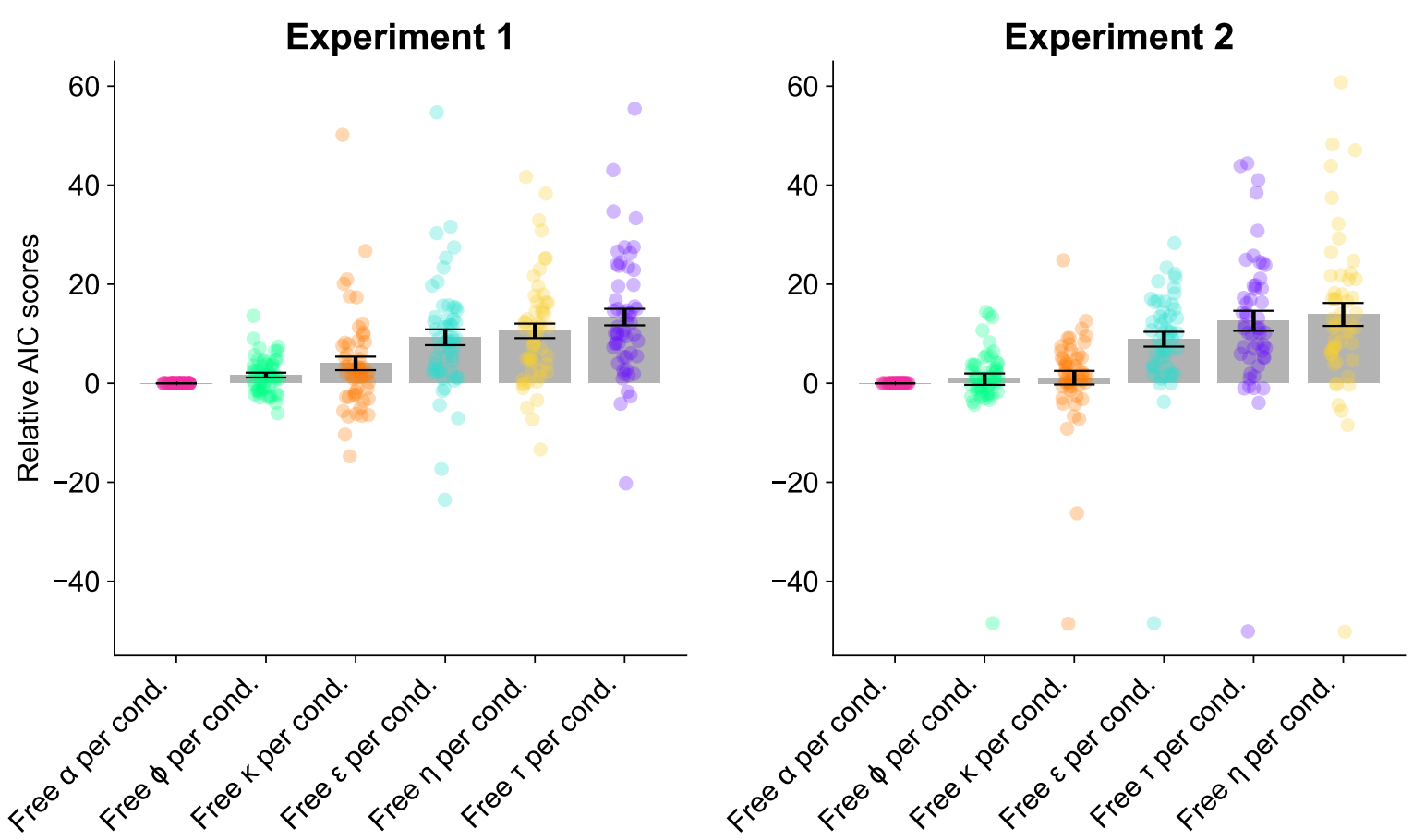

Supplementary Figure 8: **Model comparison for Experiments 1 (N = 54) and 2 (N = 51) for models with free parameters for separate Goal sub-conditions.** Bars illustrate the average AIC score relative to the best model's across all participants. Dots represent relative AIC scores for individual participants. cond = condition.

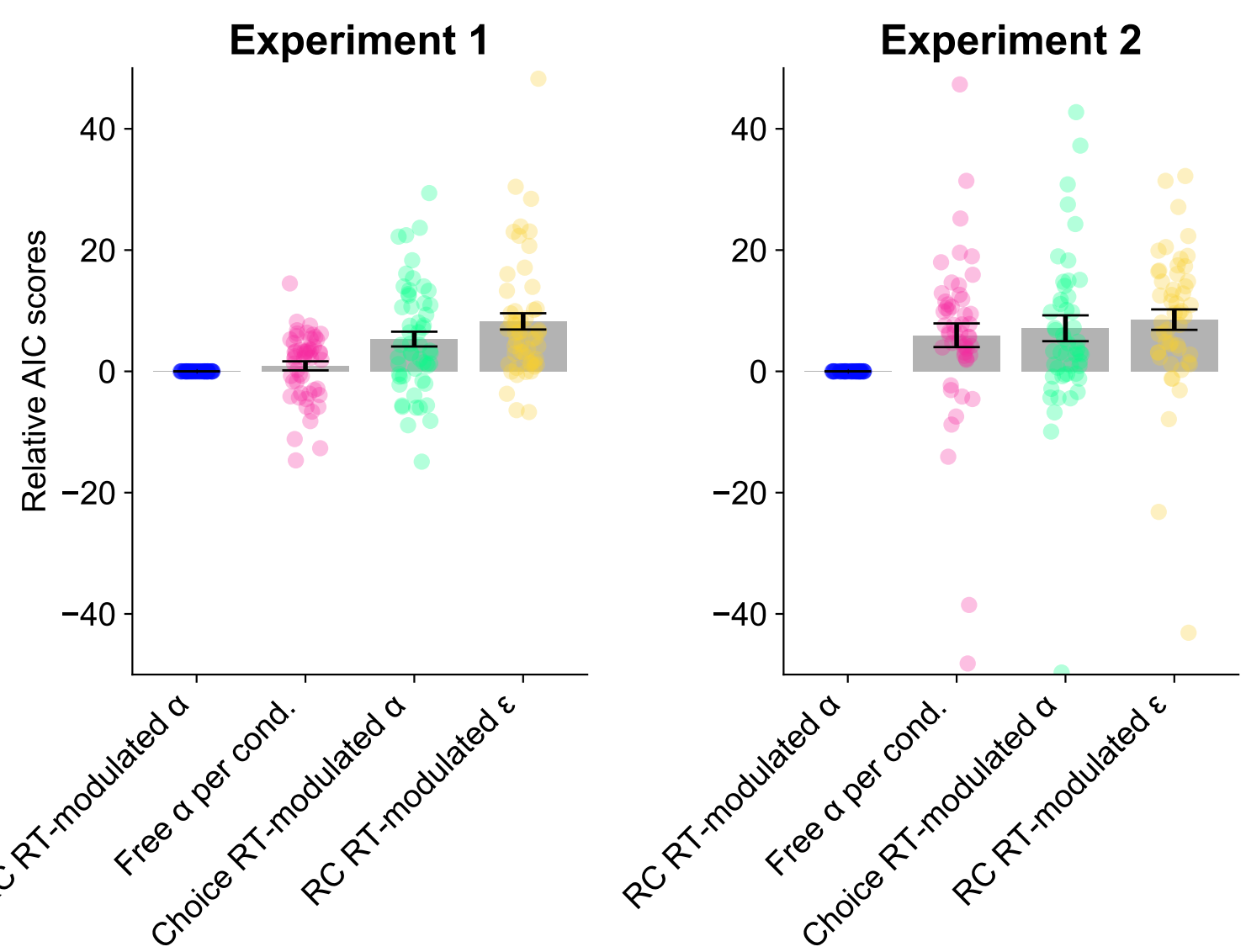

Supplementary Figure 9: **Model comparison for Experiments 1 (N = 54) and 2 (N = 51) for the main models discussed in the article.** Bars illustrate the average AIC score relative to the best model's across all participants. Dots represent relative AIC scores for individual participants. RC = reward collection. RT = reaction time.

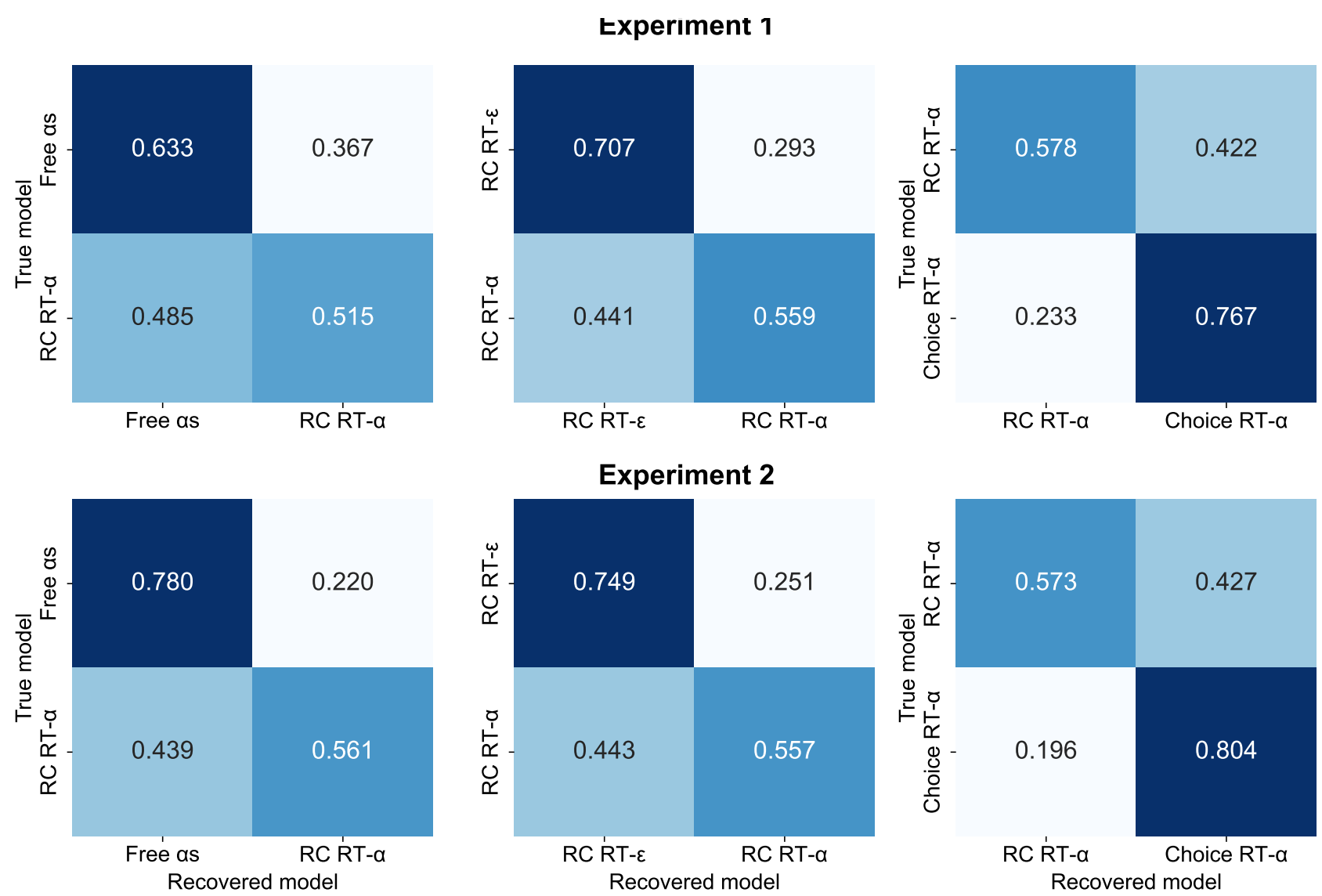

Supplementary Figure 10: **Model recovery confusion matrix.** We simulated behavior from each model 5 times per set of parameters and attempted to recover the best-fit model as measured by AIC. Each cell indicates the proportion of participants whose best-fit model corresponded to the true data-generation model. The top row illustrates results for Experiment 1, the bottom row for Experiment 2. The winning model, with learning rate α conditioned on reward collection reaction times (RC RTs; in ms) was compared to a model with free learning rates α, a model with a noise parameter ϵ, and a model with α conditioned on choice RTs, separately. In all cases, recovery of our winning model RC RT-α was more likely when data was generated from that model than the competing model.

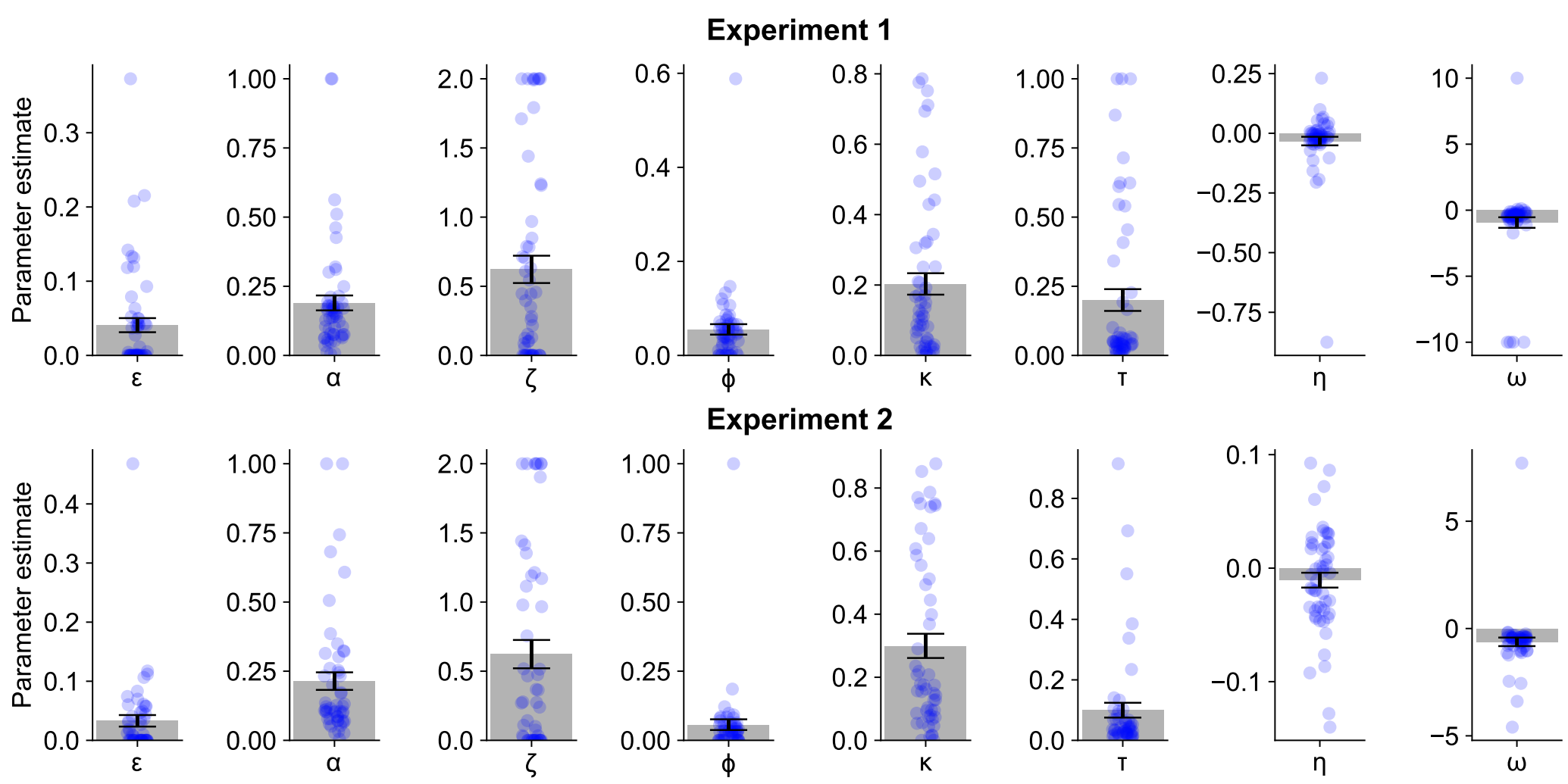

Supplementary Figure 11: **Best-fit model parameters for the winning model, with learning rate α modulated by reward collection reaction times as determined by the weighting parameter ω.** The average value of ω was significantly below zero, indicating that learning rates decreased with increased reward collection reaction times (Experiment 1: N = 54, M = -0.94 ± 0.4, W = 60, p < 0.001, r 0.80; Experiment 2: N = 51, M = -0.62 ± 0.2, W = 51, p < 0.001, r = 0.80). Bars indicate parameter estimate averages. Error bars indicate the S.E.M. Dots represent individual participants' parameter estimates.

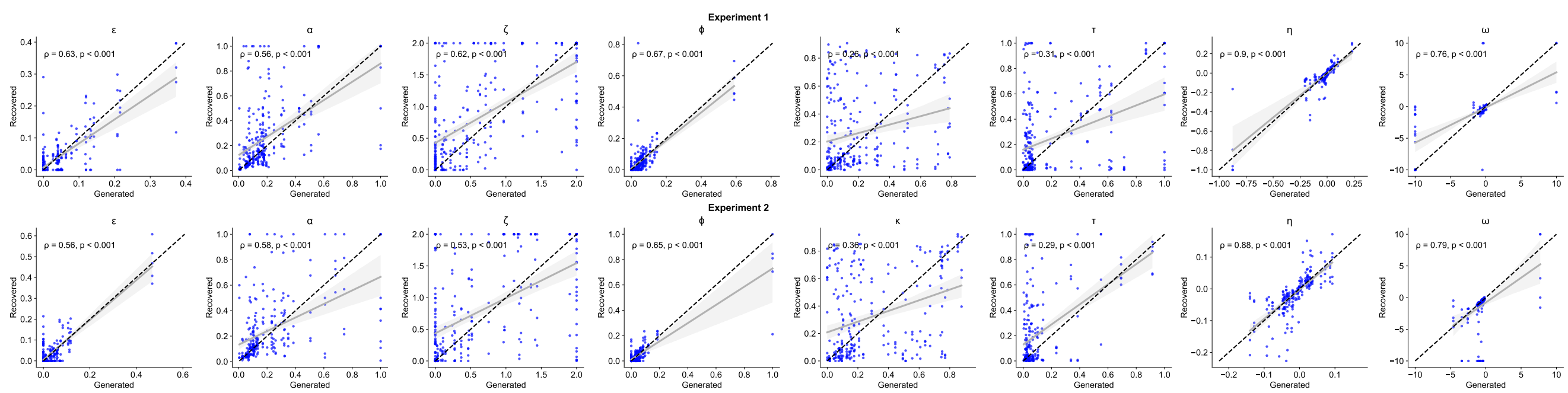

Supplementary Figure 12: **Parameter recovery for the best model in Experiments 1 (N = 54) and 2 (N = 51).** Each plot shows the correlation between a seed parameter, i.e., the best-fit parameter for each participant – used to simulate behavior – and the parameter recovered through our fitting procedure. Each parameter was used for 5 separate iterations. Dots indicate individual parameters. Spearman's ρ correlation coefficients and p-values are shown on each respective plot.

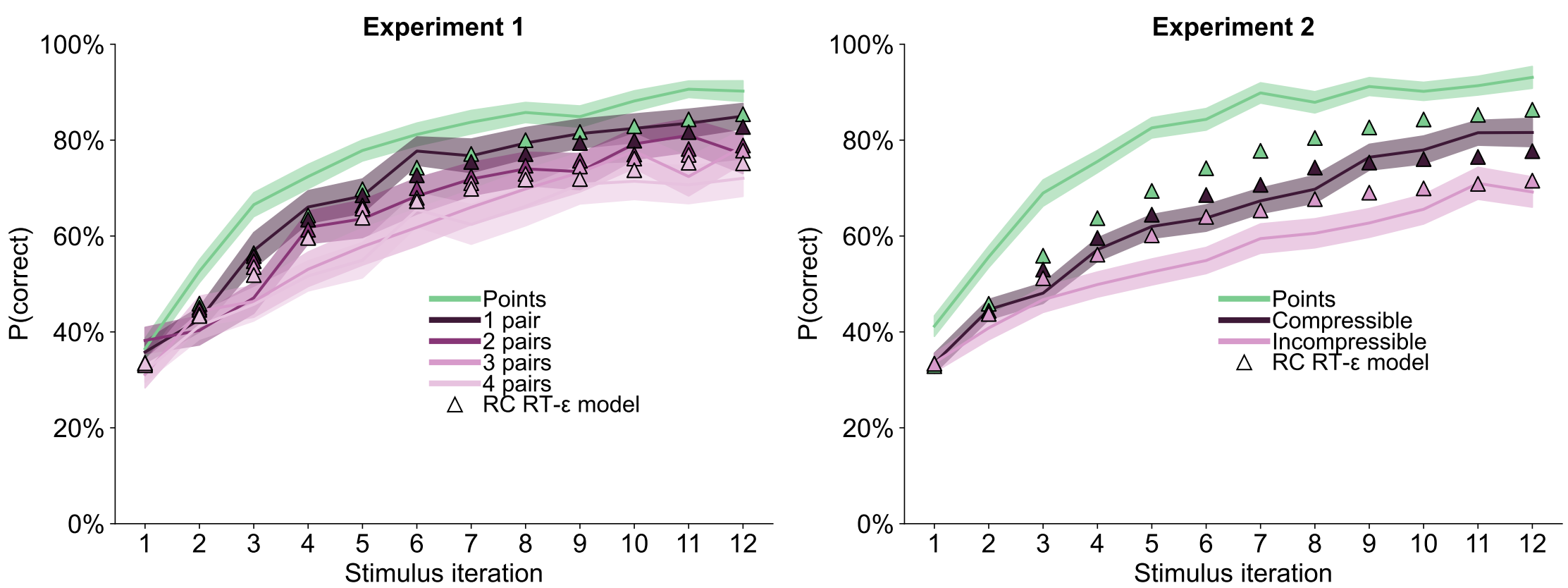

Supplementary Figure 13: **Validation for a model with ϵ (choice noise) modulated by reward collection reaction times (RTs) for Experiments 1 (N = 54) and 2 (N = 51).** Solid lines and shading indicate participants' average data and the S.E.M., triangles show model estimates.

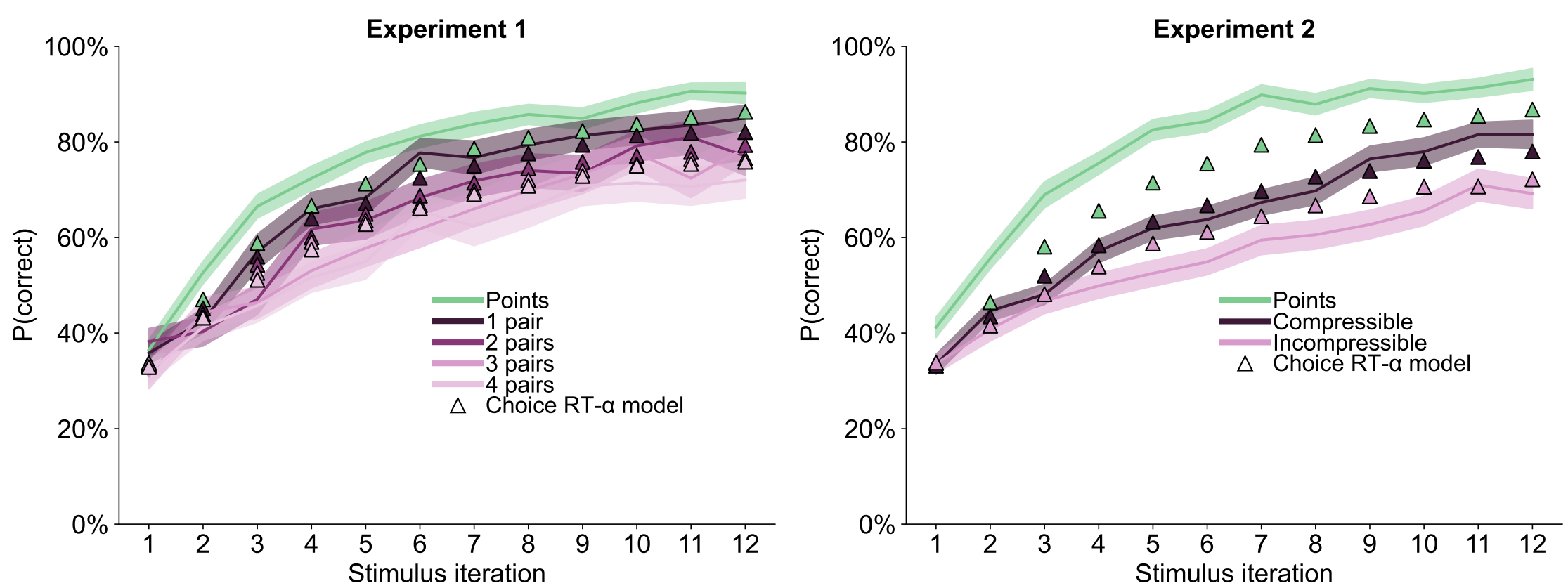

Supplementary Figure 14: **Validation for a model with α (learning rate) modulated by choice reaction times (RTs) for Experiments 1 (N = 54) and 2 (N = 51).** Solid lines and shading indicate participants' average data and the S.E.M., triangles show model estimates.

Supplementary references

[1] Molinaro, G. & Collins, A. G. Human hacks and bugs in the recruitment of reward systems for goal achievement. In *Proceedings of the Annual Meeting of the Cognitive Science Society*, vol. 45 (2023).

[2] McDougle, S. D., Ballard, I. C., Baribault, B., Bishop, S. J. & Collins, A. G. Executive function assigns value to novel goal-congruent outcomes. *Cerebral Cortex* **32**, 231–247 (2022).

[3] Gershman, S. J., Markman, A. B. & Otto, A. R. Retrospective revaluation in sequential decision making: a tale of two systems. *Journal of Experimental Psychology: General* **143**, 182 (2014).

[4] Sutton, R. S. Dyna, an integrated architecture for learning, planning, and reacting. *ACM Sigart Bulletin* **2**, 160–163 (1991).

[5] Brodeur, M. B., Dionne-Dostie, E., Montreuil, T. & Lepage, M. The bank of standardized stimuli (boss), a new set of 480 normative photos of objects to be used as visual stimuli in cognitive research. *PloS one* **5**, e10773 (2010).
