# Supplementary material for: Reward function compression facilitates goal-dependent reinforcement learning
Source: Nat Commun. 2026 Oct 8;17:11433. doi: 10.1038/s41467-026-78295-1 (PMC13737099; doi:10.1038/s41467-026-78295-1)
Supplement: Supplementary file 2 [file 41467_2026_78295_MOESM2_ESM.pdf]

nature portfolio

Corresponding author(s): Gaia Molinaro

Last updated by author(s): Aug 22, 2026

Reporting Summary

Nature Portfolio wishes to improve the reproducibility of the work that we publish. This form provides structure for consistency and transparency in reporting. For further information on Nature Portfolio policies, see our Editorial Policies and the Editorial Policy Checklist.

Statistics

For all statistical analyses, confirm that the following items are present in the figure legend, table legend, main text, or Methods section.

n/a	Confirmed	
☐	☒	The exact sample size (*n*) for each experimental group/condition, given as a discrete number and unit of measurement
☐	☒	A statement on whether measurements were taken from distinct samples or whether the same sample was measured repeatedly
☐	☒	The statistical test(s) used AND whether they are one- or two-sided<br>*Only common tests should be described solely by name; describe more complex techniques in the Methods section.*
☐	☒	A description of all covariates tested
☐	☒	A description of any assumptions or corrections, such as tests of normality and adjustment for multiple comparisons
☐	☒	A full description of the statistical parameters including central tendency (e.g. means) or other basic estimates (e.g. regression coefficient) AND variation (e.g. standard deviation) or associated estimates of uncertainty (e.g. confidence intervals)
☐	☒	For null hypothesis testing, the test statistic (e.g. *F*, *t*, *r*) with confidence intervals, effect sizes, degrees of freedom and *P* value noted<br>*Give P values as exact values whenever suitable.*
☐	☒	For Bayesian analysis, information on the choice of priors and Markov chain Monte Carlo settings
☒	☐	For hierarchical and complex designs, identification of the appropriate level for tests and full reporting of outcomes
☐	☒	Estimates of effect sizes (e.g. Cohen's *d*, Pearson's *r*), indicating how they were calculated

Our web collection on statistics for biologists contains articles on many of the points above.

Software and code

Policy information about availability of computer code

Data collection	jsPsych 6
Data analysis	We used the pingouin package (version 0.5.5) for Wilcoxon signed-rank tests, correlations, Cohen's d and r effect sizes, and Bayesian Factors, the pymer4 package (0.8.2) for mixed-effects models, and the scipy package (version 1.16) for other statistical tests. We used the pandas (version 2.3.1) and numpy (version 2.3.2) packages for data preprocessing. The fmincon function from MATLAB 2022a (via createOptimProblem and Python MATLAB engine) was used to obtain the best-fitting parameters for all computational models.

For manuscripts utilizing custom algorithms or software that are central to the research but not yet described in published literature, software must be made available to editors and reviewers. We strongly encourage code deposition in a community repository (e.g. GitHub). See the Nature Portfolio guidelines for submitting code & software for further information.

Data

Policy information about availability of data

All manuscripts must include a data availability statement. This statement should provide the following information, where applicable:

- Accession codes, unique identifiers, or web links for publicly available datasets
- A description of any restrictions on data availability
- For clinical datasets or third party data, please ensure that the statement adheres to our policy

All data is publicly available at https://doi.org/10.5281/zenodo.22069289 (GitHub repository https://github.com/gaiamolinaro/reward-function-compression).

Research involving human participants, their data, or biological material

Policy information about studies with human participants or human data. See also policy information about sex, gender (identity/presentation), and sexual orientation and race, ethnicity and racism.

Reporting on sex and gender	We report the percentage of female participants who completed the study. We do not perform any analyses by sex, gender, or other demographics. Sex and gender were not considered in the study design. Participants' sex was determined based on self-report.
Reporting on race, ethnicity, or other socially relevant groupings	n/a
Population characteristics	All participants were University undergraduates. The average age of participants in each study is reported in the manuscript.
Recruitment	All participants were recruited through our university's pool of eligible students.
Ethics oversight	The University of California, Berkeley Institutional Review Board approved the experimental procedure for all experiments (protocol #2024-04-17357)

Note that full information on the approval of the study protocol must also be provided in the manuscript.

Field-specific reporting

Please select the one below that is the best fit for your research. If you are not sure, read the appropriate sections before making your selection.

☐ Life sciences ☒ Behavioural & social sciences ☐ Ecological, evolutionary & environmental sciences

For a reference copy of the document with all sections, see nature.com/documents/nr-reporting-summary-flat.pdf

Behavioural & social sciences study design

All studies must disclose on these points even when the disclosure is negative.

Study description	Quantitative data were collected on a learning and decision-making study, in which participants learned the correct association between stimuli and keyboard keys through trial and error.
Research sample	For all experiments, we collected data from a convenience sample of University of California, Berkeley undergraduate students.<br>After exclusion, we analyzed the following:<br>Experiment 1: 54 participants (74% female, ages 18-25, M = 20.13, SD = 1.48).<br>Experiment 2: 51 participants (80% female, ages 18-32, M = 20.48, SD = 2.27).<br>Experiment 3: 9o participants (70% female, ages 18-30, M = 20.33, SD = 1.86).<br>Experiment 4: 93 participants (73% female, ages 18-27, M = 20.09, SD = 1.82).<br>Experiment 5: 51 participants (80% female, ages 18-25, M = 20.68, SD = 1.30).<br>Experiment 6: 51 participants (67% female, ages 19-26, M = 20.76, SD = 1.53).<br>Exclusion details are presented below.
Sampling strategy	A convenience sample of eligible students self-enrolled in the study via the SONA platform. Our sample was relatively uniform, consisting of university undergraduates. While our exclusion rates were typical for online studies of similar difficulty, we cannot make conclusions about the applicability of our results to less attentive participants. Appropriate sample sizes were estimated based on previous studies with similar tasks.
Data collection	Participants were recruited via SONA and completed the experiment online, on their own devices and in a location of their convenience. The experimenters were not present. We obtained informed consent from participants at the start of the study. Participants received course credit upon task completion. The experiment was coded using jsPsych and custom software. Within each experiment, all participants performed the task under the same condition. Participants received minimal information about the scope of the experiment. Blinding was not applicable.
Timing	Data for this experiment were collected between 9/2022 and 3/2024
Data exclusions	To reduce spurious effects due to inattentive participants, exclusion criteria were devised based on visually identified elbow points Xia et al., 2021). Participants were excluded if they: 1) failed to respond on more than 25 trials in total, 2) missed more than 10 trials consecutively, 3) selected the same response more than 15 times in a row, 4) failed to accurately collect rewards in Points trials, as evidenced by either a d' below or equal to 1, or more than 20 reward collection errors, 5) did not understand the instructions for reward collection in Goals trials, as evidenced by a reward collection d' below or equal to 0, or more than 50 reward collection errors in the Goals condition, 6) failed to collect rewards entirely in one or more blocks, 7) had an average response time of less than 300 ms in either the Points or the Goals condition (or both). Participants under one or more such categories were excluded from further analysis.<br>Experiment 1: Sixty-seven participants completed Experiment 1, 12 of whom did not match our inclusion criteria. We analyzed data from the remaining 54 participants.

	Experiment 2: Seventy-one participants completed Experiment 2. After excluding 20 who failed to meet our inclusion criteria, we analyzed data from the remaining 51 participants.<br>Experiment 3: One hundred and twenty-five participants completed Experiment 3. Thirty-five participants failed to meet baseline requirements and were excluded from further analysis. Therefore, data from 90 participants were analyzed.<br>Experiment 4: One hundred and twenty-one participants completed Experiment 4. The final data set after exclusion comprised 93 participants.<br>Experiment 5: Sixty-seven participants completed Experiment 5, 16 of whom did not meet our exclusion criteria. Data from 51 subjects were analyzed.<br>Experiment 6: Seventy-one participants completed Experiment 6, 20 of which did not pass our exclusion criteria. Data from 51 subjects were analyzed.
Non-participation	Because participants self-enrolled, no participant declined the invitation.
Randomization	Data were collected independently for each experiment. Participants who had completed one experiment were ineligible for any of the others. The exact sequence of stimuli was pseudorandomized across participants. Within each experiment, all participants performed the task under the same condition.

Reporting for specific materials, systems and methods

We require information from authors about some types of materials, experimental systems and methods used in many studies. Here, indicate whether each material, system or method listed is relevant to your study. If you are not sure if a list item applies to your research, read the appropriate section before selecting a response.

Materials & experimental systems

n/a	Involved in the study	
☒	☐	Antibodies
☒	☐	Eukaryotic cell lines
☒	☐	Palaeontology and archaeology
☒	☐	Animals and other organisms
☒	☐	Clinical data
☒	☐	Dual use research of concern
☒	☐	Plants

Methods

n/a	Involved in the study	
☒	☐	ChIP-seq
☒	☐	Flow cytometry
☒	☐	MRI-based neuroimaging

Plants

Seed stocks	n/a
Novel plant genotypes	n/a
Authentication	n/a
