# Supplementary material for: Reward function compression facilitates goal-dependent reinforcement learning
Source: Nat Commun. 2026 Oct 8;17:11433. doi: 10.1038/s41467-026-78295-1 (PMC13737099; doi:10.1038/s41467-026-78295-1)
Supplement: Supplementary file 3 [file 41467_2026_78295_MOESM3_ESM.pdf]

nature portfolio

Peer Review File

Reward function compression facilitates goal-dependent reinforcement learning

Corresponding Author: Dr Gaia Molinaro

This file contains all reviewer reports in order by version, followed by all author rebuttals in order by version. Parts of this Peer Review File have been redacted as indicated to remove third-party material.

Version 0:

Reviewer comments:

Reviewer #1

(Remarks to the Author)
This study investigates the cognitive mechanisms by which humans efficiently process and act upon multiple coexisting goals — a capacity that is essential in complex environments. The authors propose a conceptual model in which goal-dependent reinforcement learning is initially supported by working memory, but becomes more efficient once a "compressed reward function" is extracted and transferred to long-term storage. They examine this process across two main and four supplementary behavioral experiments, complemented by computational modeling of the behavioral data.

I found the motivation of this work interesting, and the experiments and findings have some value. At the same time, the experiments and the computational model do not, in my view, directly support or instantiate the conceptual model proposed by the authors (or at least, the relationship between the two was not clear to me), and the authors' claims do not appear to be as clearly supported as the manuscript suggests. My specific concerns and suggestions are described below. I may not yet fully understand all aspects of the manuscript, so I would welcome clarification if I have misunderstood any of these points.

First, the process of "compression" of the reward function does not appear to be explicitly addressed in either the experiments or the computational model. The process of "transfer to long-term memory storage" is included in the conceptual model (Figure 1), but I do not see this process as being adequately supported by the experimental data.

In particular, the process at work in Experiment 2 might be better described as feature selection rather than goal compression. For instance, the feature-based attention learning process discussed by Niv et al. (2015; https://pubmed.ncbi.nlm.nih.gov/26019331/) seems relevant here (with the difference that, in the present study, the relevant features belong to the goal rather than to the choice options, as in Niv et al.). Under this interpretation, it is understandable that identifying the relevant feature would reduce the working memory load associated with maintaining the goal. However, it does not directly follow that a reward function is being maintained in long-term memory.

The authors appear to treat reward collection RT as a behavioral marker of compression. However, the underlying logic — "compression leads to automatization," "automatization shortens RT," and therefore "shorter RT is evidence of compression" — seems somewhat circular. As an alternative, one could imagine that as learning progresses, the stimulus-response pairs become habitual and no longer require working memory, which in turn frees up working memory to be used for goal maintenance. The model does include a habit component (C value) that captures the habitization process, but the resulting release of working memory is not represented in the model. The Discussion does explicitly acknowledge the limitations regarding the causal relationship between RT and performance, but this limitation nonetheless feels like a weakness of the study.

Regarding the computational model, the process of reward function compression is not explicitly modeled, and the model does not correspond to the conceptual model proposed by the authors. In this sense, the potential of computational modeling does not seem to be fully realized. To represent the process of reward function compression, one possible approach would be to assume that the feedback r — currently treated as binary — changes over the course of learning, and/or takes different values across conditions. (Mathematically, under certain conditions, this is equivalent to the inverse temperature β changing; however, in the present study β is fixed at 25 and is shared across conditions.)

(Remarks on code availability)

Reviewer #2

(Remarks to the Author)
This is a rigorous and intriguing paper that I believe makes a substantial contribution to the field and is suitable for publication in Nature Communications. The authors propose a framework for understanding how humans flexibly attribute value to arbitrary outcomes in the environment which I believe is an under-explored phenomenon in reinforcement learning settings (which have tended to focus on learning well-defined external rewards). I am very sympathetic to the idea that this flexibility in our reward function capacity is fundamental to our capacity to learn such a diversity of tasks, and I appreciate the authors' attempt to address this within the reinforcement learning literature.

The authors' suggestion is that this process of flexible attribution of reward to arbitrary outcomes relies on an initial working memory process that is costly. Over time this costly recruitment of WM is replaced (through compression) by long-term memory of the goal value, freeing up working memory for other processes. The authors leverage a creative task design to explore this, and present a number of studies to address different explanations. While I believe the article is suitable for publication, I have a few remaining concerns below to be addressed.

1. Compression vs. Repetition claims
While I am sympathetic to the overall framework, I'm not entirely convinced by the specific claim that goal compression is necessary to transition from a costly WM representation to long-term memory, and specifically their strong claims that repetition alone is insufficient. I think this would benefit either from further analysis, or weakening the strength of these claims and discussion of alternative explanations.

a) I believe the compressible vs. incompressible goals result in Experiment 2 may be partially explained by active interference rather than lack of compression per se in the incompressible condition. In the incompressible condition, no single stimulus dimension reliably predicts goal status, meaning that participants who attempt to learn over individual features (a strategy that has been demonstrated in attention-modulated reinforcement learning paradigms e.g. Leong et al., 2017) will encounter active conflict between dimensions. This is importantly different from simply failing to compress because it's a situation in which partial learning actively misleads. Whether or not one frames this as a failure of compression, the mechanism may be better described as dimensional interference rather than a working memory load effect. The fact that the learning curves and reward collection rate in the Incompressible condition in Experiment 2 look similar to those in the 'Conflicting' condition in Experiment 6 (which is explicitly designed to test interference from reversing the goals), to me seems like further evidence for this alternative interpretation.

b) Relatedly, and importantly, participants *do* improve over stimulus iterations even in the incompressible and conflicting conditions, which sits in tension with the strong claim that "re-experiencing the same goal more times" is irrelevant to performance if compression is not possible. If pure repetition truly conferred no benefit, we might not expect to see this within-condition improvement at all. I suggest the claims about repetition as insufficient for learning the goal values be weakened.

c) The most compelling evidence for the claim that repetition alone is not enough for goal learning is Experiment 5, which I found the most surprising result in the paper, and which I would suggest deserves a place in the main manuscript rather than the supplementary materials if the authors want to keep this claim. However, I also think this could be better tested by fitting a model to the Experiment 5 data in which separate learning rates are used for trials where the outcomes are the repeated-goal versus novel-goal images within a block. If repeated goals truly confer no benefit for learning, the dual learning rate model should not outperform the baseline with a single learning rate. Otherwise I worry that averaging performance over conditions is obscuring a real repetition effect that the current analyses cannot detect.

2. Process of goal learning
While I don't think this is necessary for the study to be published, one limitation of the paper is that the authors offer no model for the process of goal learning itself (by which reward in WM is transferred to memory), through compression or otherwise. I do appreciate the author's usage of reward collection RT as a proxy for compression efficiency, but the mechanism by which goal features are consolidated is left unspecified. One could imagine, for example, a model in which learners incrementally weight stimulus dimensions by their association with Goal, with the effective learning rate for the stimulus-response association on any given trial scaled by the strength of this goal representation. I do not think this model is required for publication, but I do think it would improve the paper to have a discussion of this process, or for the authors to more explicitly acknowledge this as a limitation, particularly given that the current RT models underestimate the condition differences (e.g. Figure S12 and S13), and that the mechanistic claims in the discussion are stated with some confidence.

3. Reward collection accuracy as a control
The authors use reward collection d' as a check that goal recognition failures do not explain the learning impairments for arbitrary goals, and argue that the absence of a correlation with learning performance rules this out. I would suggest a more stringent approach on two grounds. First, d' collapses across false positives and false negatives, which may have quite different implications for learning. A participant who frequently collects non-goal outcomes is in a different situation to one who frequently misses goal outcomes, and these should be examined separately within the correlations. Second, beyond the correlation analysis, I would suggest including reward collection accuracy components directly as covariates in the mixed effects models predicting learning performance, for example:

learning accuracy ~ condition + stimulus_iteration + block_number + stimulus_d' + (1|participant)

Where stimulus_d' could be the reward collection accuracy for that particular stimulus within that block, for example. The authors might have better ways of controlling for this, but I think it is important to control for these errors more explicitly within the regression when quantifying the impact of goal condition on learning.

Minor points
1. A brief definition of d' as the measure of reward collection accuracy (I assume this is z-scored hit rate minus z-scored false alarm rate) would improve accessibility for readers less familiar with signal detection theory.
2. There is a cut-off sentence at the end of the Experiment 5 supplementary text.
3. The task figure could be improved by adding a clear visual separator to emphasise that the goal/nongoal images are entirely independent of the stimulus-response mapping. This took some time for me to parse and is central to understanding the design. It might also be nice to illustrate the stimulus-response associations themselves i.e. picture-keypress to understand that the fruit/veg are the pictures being associated with keys, and the bottom images are the pictures that form the goal.
4. If I've understood the parameters correctly in Figure S10, the response stickiness parameter η is always negative. This seems like the opposite of what is normally found in RL and I'm wondering if there there is explanation for this. One possibility is that the task structure discourages perseveration for example, if the same picture is never shown twice consecutively and if pictures are always associated with different keys, participants may adopt a habitual switch strategy. If so, it would be helpful to note as it has implications for interpreting the model.

(Remarks on code availability)
I couldn't see a code base or availability statement linked in the text but there is sufficient detail in the methods

Reviewer #3

(Remarks to the Author)
This paper investigates whether people learn more slowly when they need to maintain and learn larger sets of goals, compared to situations in which (1) fewer goals are used, (2) goals can be compressed or grouped, or (3) learning can rely on overtrained reward goals such as point systems. The review was written together with a postdoc from the lab. Overall, we found much to like about this work. The studies are well-powered, carefully controlled, and address a fundamental question about the representational constraints underlying learning. Although some of the findings appear quite intuitive, we are not aware of previous studies that systematically mapped out these principles in such a way. As such, the empirical work, together with the modelling, may potentially provide a solid foundation for future work investigating goal-based learning. However, at the same time, we thought the modelling was only briefly motivated and did not really evaluate some of the central theoretical claims that motivated the paper:

Most notably, throughout the manuscript the authors repeatedly emphasize the role of working memory constraints in goal learning and goal compression. In addition, the senior author has previously published highly relevant work on the interaction between reinforcement learning and working memory. For this reason, we were surprised that none of the many models that were compared included an explicit working memory component? While reading the paper, we assumed that one of the core hypotheses under investigation was that larger goal spaces impose greater demands on working memory during reinforcement learning. However, the models compared in the current manuscript do not directly test this hypothesis. Similarly, the idea of goal compression is not really formalized in the modelling. Instead, the modelling did include a Hebbian-learning component, following the recent paper by Collins (2026, Nature Human Behaviour). However, even in that work, the proposed framework still included a working memory component as well, whereas the current model comparisons do not. We therefore believe the paper should either (a) incorporate and directly evaluate a working memory component, or (b) clearly clarify why the current modelling approach does not require this.

Second, we wondered why the model comparison systematically allowed block-specific variation in most model parameters, but not in parameters such as tau or the forgetting rate. Ideally, these parameters should also be allowed to vary across blocks and be included in the comparison procedure.

Finally, although we believe the central question is fundamentally interesting from a cognitive science perspective, the broader relevance of the work would benefit from the inclusion of a few concrete everyday examples that map closely onto the current paradigm. Providing two or three such examples would help readers connect the experimental manipulations to naturally occurring forms of learning and goal management.

A very minor point concerns the relation to the action–effect learning literature. We do not believe this literature threatens the novelty of the present work. Nevertheless, some brief discussion of these studies may help situate the manuscript more broadly within existing research on action–outcome representations and learning. The following papers in particular came to mind:
Wolfensteller, U., & Ruge, H. (2011). On the timescale of stimulus-based action–effect learning. Quarterly Journal of Experimental Psychology, 64(7), 1273-1289.
Janczyk, M., Eichfelder, L., Liesefeld, H. R., & Franz, V. H. (2024). Learning and transfer of response–effect relations. Quarterly Journal of Experimental Psychology, 17470218241284259.

(Remarks on code availability)

Version 1:

Reviewer comments:

Reviewer #1

(Remarks to the Author)
I appreciate the authors' clear and thoughtful responses to my comments, and the corresponding revisions to the manuscript. While the formal modeling of the goal compression process is deferred to future work — which is a somewhat modest resolution — I recognize that the present study still offers value in establishing the phenomenon and articulating the conceptual framework.

(Remarks on code availability)

Reviewer #2

(Remarks to the Author)
I thank the authors for their thorough and thoughtful responses, which have addressed nearly all of my concerns. I'm satisfied with the revisions regarding the claims about null repetition effects, the conceptual framing of the models, and all the minor points. I think the task figure is also now very clear.

I have one remaining request on point 3 i.e. the analyses of incorrect reward collection responses to check the results aren't confounded by confusion about the current goal. While I appreciate the pointer to the trial-by-trial modeling result in Molinaro & Collins (2023), that analysis concerns Experiment 3, whereas my concern is more important for the main experiments, particularly Experiment 2, where reward collection accuracy did differ significantly between conditions. Since the authors already have the relevant trial-level data and mixed-effects models, I think it should be pretty quick to run a control regression like the one I suggested (for example: learning accuracy ~ condition + stimulus iteration + block number + stimulus-level collection accuracy + (1|participant)), or an equivalent specification of the authors' choice e.g. separating the false positives from false negatives within the regression. I expect this will confirm all the existing conclusions, and including it would make the central results feel more robust. With this addition, I'm happy to recommend publication.

(Remarks on code availability)

Reviewer #3

(Remarks to the Author)
We would like to thank the authors for engaging with our comments. While we still think a slightly more elaborate modelling approach to support some of the authors' central claims (e.g., about goal compression) would be desirable, we also think the present behavioral findings are interesting and already provide a good foundation for future work investigating goal-based learning.

(Remarks on code availability)

Version 2:

Reviewer comments:

Reviewer #2

(Remarks to the Author)
Many thanks to the authors for engaging with my request for a further control analysis regarding goal identification errors. The mixed effects regression they propose looks very sensible, and I appreciate their choice to split up false negatives and false positives. I think the manuscript is now very strong, and I am very happy to recommend this for publication.

(Remarks on code availability)

Open Access This Peer Review File is licensed under a Creative Commons Attribution 4.0 International License, which permits use, sharing, adaptation, distribution and reproduction in any medium or format, as long as you give appropriate credit to the original author(s) and the source, provide a link to the Creative Commons license, and indicate if changes were made.
In cases where reviewers are anonymous, credit should be given to 'Anonymous Referee' and the source.
The images or other third party material in this Peer Review File are included in the article's Creative Commons license, unless indicated otherwise in a credit line to the material. If material is not included in the article's Creative Commons license and your intended use is not permitted by statutory regulation or exceeds the permitted use, you will need to obtain permission directly from the copyright holder.
To view a copy of this license, visit https://creativecommons.org/licenses/by/4.0/

We thank all reviewers for their constructive, supportive feedback, which we have addressed through comments and changes to the manuscript. To facilitate the discussion, we report the reviewers' comments verbatim, in black. Our responses are in blue, with text from the updated manuscript written in blue and indented. *Italics* are used to highlight new edits to existing sentences or paragraphs. For convenience, in-text citations are reported in APA style in this document, even though the manuscript uses numbered citations. Please also note that some changes have been made to comply with editorial policies (e.g., shortening the abstract, submitting the supplements as a separate PDF). A version of the manuscript tracking all changes from the initial submission is attached.

Reviewer #1

This study investigates the cognitive mechanisms by which humans efficiently process and act upon multiple coexisting goals — a capacity that is essential in complex environments. The authors propose a conceptual model in which goal-dependent reinforcement learning is initially supported by working memory, but becomes more efficient once a "compressed reward function" is extracted and transferred to long-term storage. They examine this process across two main and four supplementary behavioral experiments, complemented by computational modeling of the behavioral data.

I found the motivation of this work interesting, and the experiments and findings have some value. At the same time, the experiments and the computational model do not, in my view, directly support or instantiate the conceptual model proposed by the authors (or at least, the relationship between the two was not clear to me), and the authors' claims do not appear to be as clearly supported as the manuscript suggests. My specific concerns and suggestions are described below. I may not yet fully understand all aspects of the manuscript, so I would welcome clarification if I have misunderstood any of these points.
Thank you for your thorough assessment of our manuscript. Below, we address specific concerns and offer clarifications.

First, the process of "compression" of the reward function does not appear to be explicitly addressed in either the experiments or the computational model. The process of "transfer to long-term memory storage" is included in the conceptual model (Figure 1), but I do not see this process as being adequately supported by the experimental data.
The reviewer correctly pointed out that we do not establish formal models of compression or long-term memory transfer. While we intend to do so in future work, the current paper focuses on establishing a phenomenon, proposing a conceptual model that can guide follow-up studies, and providing initial evidence in support of the latter. For this reason, we refer to our model as conceptual and explicitly state that algorithmic details are not

provided. To make this point even clearer to readers, we have edited our text in several locations (italics for emphasis here only).

In the Abstract, we now say:

> *Although the algorithmic details remain to be established*, our behavioral results and computational models suggest that efficient goal-directed learning relies on compressing complex goal information into a stable reward function.

In the Introduction, we state:

> In our *conceptual* model, a working memory (WM) component enables us to instantly attribute goal-contingent rewarding properties even to abstract, novel goals. [...] While we remain agnostic with regard to its algorithmic implementation in the brain *and formal instantiations of reward function compression itself*, our model makes specific predictions, which we test in this study.

In Figure 1's caption, we specify:

> *In our proposed conceptual model*, a working memory (WM) component enables us to instantly attribute goal-contingent rewarding properties even to abstract, novel goals.

In the computational modeling section:

> Treating reward collection RTs as proxies for reward processing efficiency (*potentially gained through long-term memory transfer*), we also built a family of models where one of the parameters was directly influenced by the block-wise median (log) reward collection RT for the corresponding condition (i.e., Points, or a specific Goals sub-condition; see Materials and Methods for details) to a degree defined by parameter ω.

In the Discussion, in three separate paragraphs:

> Through a series of experiments and computational modeling, we found ~~strong~~ support for this framework. [...] *However, a formal model of reward functions' long-term memory transfer remains to be established.* [...] Furthermore, the process by which people create compressed reward functions from raw example outcomes – *which we did not model in detail* – remains to be elucidated.

In particular, the process at work in Experiment 2 might be better described as feature selection rather than goal compression. For instance, the feature-based attention learning process discussed by Niv et al. (2015; https://pubmed.ncbi.nlm.nih.gov/26019331/) seems relevant here (with the difference that, in the present study, the relevant features belong to the goal rather than to the choice options, as in Niv et al.). Under this interpretation, it is understandable that identifying the relevant feature would reduce the working memory load associated with maintaining the goal. However, it does not directly follow that a reward function is being maintained in long-term memory.

We agree that feature selection on goals, rather than choice options, likely has a role in Experiment 2. However, we see it as the premise of our account, not as an alternative

explanation. Niv et al. (2015) describe a process that might explain how a compressed reward function is formed; our claim, instead, concerns what happens once it is established. While we do not directly probe long-term memory transfer, we do observe that performance improves gradually over time, which would be less likely if participants had sudden insights about relevant features. More importantly, if performance gains were due to a general WM load reduction, they should correlate with both choice and reward collection RTs. Instead, we only observe the latter, and our modeling results linking RTs to learning rates confirm that WM load reduction specifically benefits reward processing, not choice. We now cite Niv et al., 2015, in the Discussion:

> Several mechanisms might support reward function compression, including attention-based feature selection Niv et al., 2015; Leong et al., 2017).

The authors appear to treat reward collection RT as a behavioral marker of compression. However, the underlying logic — "compression leads to automatization," "automatization shortens RT," and therefore "shorter RT is evidence of compression" — seems somewhat circular. As an alternative, one could imagine that as learning progresses, the stimulus-response pairs become habitual and no longer require working memory, which in turn frees up working memory to be used for goal maintenance. The model does include a habit component (C value) that captures the habitization process, but the resulting release of working memory is not represented in the model. The Discussion does explicitly acknowledge the limitations regarding the causal relationship between RT and performance, but this limitation nonetheless feels like a weakness of the study.

Regarding circularity, we note that we model participants' *choices* with respect to stimulus-action associations, but RTs come from the separate behavior of *reward collection* at each trial's outcome, averaged across blocks, which obviates the circularity. The logic underlying our approach is: 1) short RTs are evidence of efficient outcome processing; 2) efficient outcome processing is predictive of faster, more robust learning that follows the direction predicted by the experimental manipulation; 3) such performance gains can be interpreted as a decrease in the use of a shared resource between reward processing and associative learning. After realizing this was not sufficiently clear in our draft, we added the following text immediately after introducing our model linking reward collection RTs and learning parameters:

> This model design rests on the assumption that faster RTs indicate more efficient processing and that, if reward-processing gains free up shared resources, they should relate to specific benefits in learning. In contrast, if learning improvements were unrelated to reward processing, linking the two systems would not capture behavior more accurately than a model with parameters independent of reward collection RTs.

While the alternative the reviewer suggested is valid, we do not find support for it in our data. In Experiment 2, each participant completed two blocks of each condition; the

Goal/Nongoal images of each condition were repeated across the two rounds, while the response stimuli were new in each block; therefore, the second set of Goals blocks required new stimulus-response learning, preventing habitual stimulus-action choices by design. Yet, reward collection became more efficient in the second block of the Compressible condition compared to the first, which does not match the causal direction suggested by the reviewer. We now report this observation in the main text and show it in the supplementary materials:

> Moreover, despite new learning, reward collection RTs decreased from the first set of Goals blocks ($M = 422.18 \pm 13.92$) to the second (where goal/nongoal images were reused; $M = 316.06 \pm 15.9$, $t(50) = 8.0$, $p < 0.001$). This RT decrease was sharper for the Compressible, compared to the Incompressible condition: a linear mixed-effects model predicting reward collection RTs showed a significant interaction between Goals condition type and experiment phase (early vs. last; $\beta = -13.58$ +± 6.14, 95% CI = [-25.62, -1.53], $p < 0.001$), controlling for stimulus iteration and its interaction with condition type. Similar findings applied to learning performance increases across phases (early learning accuracy $M = 0.53 \pm 0.02$, late learning accuracy $M = 0.66 \pm 0.02$; $t(50) = -9.45$, $p < 0.001$; Goals condition type by experiment phase interaction $\beta = -0.03$ +± 0.01, 95% CI = [0.01, 0.04], $p < 0.001$). Together, these results suggest that learning benefits could not be fully attributed to more habitual choice behavior (Supplementary Fig. 2).

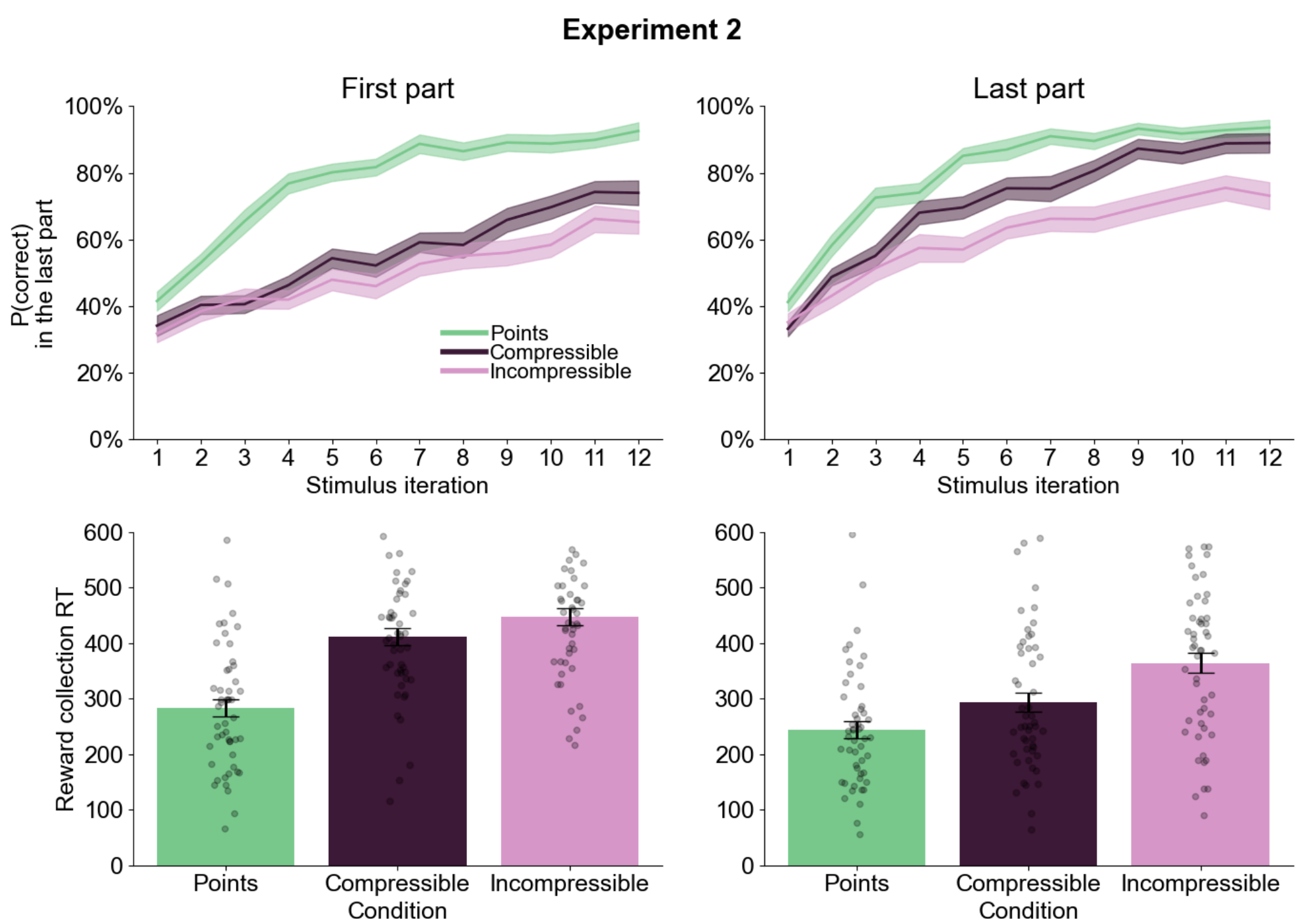

Supplementary Figure 2: Learning curves and reward collection RTs for Experiment 2's first and last (right) set of blocks. Bars indicate participants' average behavior, dots show individual participants' scores. Error bars indicate the S.E.M.

Regarding the computational model, the process of reward function compression is not explicitly modeled, and the model does not correspond to the conceptual model proposed by the authors. In this sense, the potential of computational modeling does not seem to be fully realized. To represent the process of reward function compression, one possible approach would be to assume that the feedback r — currently treated as binary — changes over the course of learning, and/or takes different values across conditions. (Mathematically, under certain conditions, this is equivalent to the inverse temperature β changing; however, in the present study β is fixed at 25 and is shared across conditions.)
We agree that the present manuscript does not offer a formal quantitative model of reward function compression. In other work, we are actively pursuing the possibility that reward feedback is subjective and dynamic (Molinaro & Collins, *CogSci*, 2023; Pan et al., in prep.). Here, we avoid modeling reward function compression directly for two reasons. First, none of the experiments directly focus on probing its dynamics; therefore, any computational model we provided would not be directly testable with the data, but would need follow-up verification, which we believe is better left for separate work. We contend that the current manuscript represents an important step, using existing data and models, toward establishing a previously unknown phenomenon and a theoretical proposal. Second, our conceptual model and data support the idea that performance gains are not due to reward function compression per se, but to interactions between WM and other learning components that arise as a result; treating reward feedback as subjective would not, by itself, sufficiently capture this dynamic. In response to this and the reviewer's first point, we made several edits to clarify that the present contribution does not provide an algorithmic model of reward function compression itself – please see our reply to the first comment above for details. Additionally, we note that a subjective, dynamically varying reward would not be equivalent to a changing β: β is applied to Q-values, which integrate the past reward history, rather than the current reward only. The timeframe would not directly match, unless the reviewer refers to a β applied to reward collection, which is not what we model.

Reviewer #2

This is a rigorous and intriguing paper that I believe makes a substantial contribution to the field and is suitable for publication in Nature Communications. The authors propose a framework for understanding how humans flexibly attribute value to arbitrary outcomes in the environment which I believe is an under-explored phenomenon in reinforcement learning settings (which have tended to focus on learning well-defined external rewards). I am very sympathetic to the idea that this flexibility in our reward function capacity is

fundamental to our capacity to learn such a diversity of tasks, and I appreciate the authors' attempt to address this within the reinforcement learning literature.

The authors' suggestion is that this process of flexible attribution of reward to arbitrary outcomes relies on an initial working memory process that is costly. Over time, this costly recruitment of WM is replaced (through compression) by long-term memory of the goal value, freeing up working memory for other processes. The authors leverage a creative task design to explore this, and present a number of studies to address different explanations. While I believe the article is suitable for publication, I have a few remaining concerns below to be addressed.
We thank the reviewer for such a positive review and for their thoughtful comments, which we address below.

1. Compression vs. Repetition claims
While I am sympathetic to the overall framework, I'm not entirely convinced by the specific claim that goal compression is necessary to transition from a costly WM representation to long-term memory, and specifically their strong claims that repetition alone is insufficient. I think this would benefit either from further analysis, or weakening the strength of these claims and discussion of alternative explanations.
a) I believe the compressible vs. incompressible goals result in Experiment 2 may be partially explained by active interference rather than lack of compression per se in the incompressible condition. In the incompressible condition, no single stimulus dimension reliably predicts goal status, meaning that participants who attempt to learn over individual features (a strategy that has been demonstrated in attention-modulated reinforcement learning paradigms e.g. Leong et al., 2017) will encounter active conflict between dimensions. This is importantly different from simply failing to compress because it's a situation in which partial learning actively misleads. Whether or not one frames this as a failure of compression, the mechanism may be better described as dimensional interference rather than a working memory load effect. The fact that the learning curves and reward collection rate in the Incompressible condition in Experiment 2 look similar to those in the 'Conflicting' condition in Experiment 6 (which is explicitly designed to test interference from reversing the goals), to me seems like further evidence for this alternative interpretation.
While we appreciate this concern, Experiment 2's Incompressible condition and Experiment 6's conflicting condition are not directly comparable because the former was isolated within condition-specific blocks, while the latter was part of a within-block manipulation. However, a test for the active conflict hypothesis presented by the reviewer can be performed by comparing the Incompressible Goals condition from Experiment 2 to the 4-goal condition of Experiment 1, where goal/nongoal images were widely different

fractal images not characterized by directly conflicting features. We reported this analysis in the Supplements and reproduce here for convenience:

> By contrast, learning performance in the last block of the Incompressible Goals conditions (0.61 ± 0.03) remained lower ($t(142) = 3.07$, $p = 0.003$), resembling performance in the four-pair Goals condition of Experiment 1 instead ($t(103) = -0.55$, $p = 0.580$, $BF_{10} = 0.24$, showing moderate evidence for the null hypothesis).

Because performance in Experiment 2's Incompressible condition was similar to that of a condition with 4 goals and no feature overlap between goals, this result speaks against the active interference hypothesis. We acknowledge, however, that some active interference is still possible. In any case, we find the dimensional interference interpretation to still be in line with our general message: that gradually acquired reward functions enable flexible goal-dependent learning. To make this point clearer, we add a broadening sentence in the Discussion:

> This suggests that performance improvements are not merely a function of repeated exposure to the same goals, but rather depend on the ability to extract a simple, generalizable reward function – especially when the goals themselves are too complex to be fully stored in WM. *Several mechanisms might support reward function compression, including attention-based feature selection* Niv et al., 2015; *Leong et al.*, 2017). *While this specific study allowed for associations between success and nameable rules, similar processes might apply to differently learned reward functions, which may or may not require compression.*

b) Relatedly, and importantly, participants *do* improve over stimulus iterations even in the incompressible and conflicting conditions, which sits in tension with the strong claim that "re-experiencing the same goal more times" is irrelevant to performance if compression is not possible. If pure repetition truly conferred no benefit, we might not expect to see this within-condition improvement at all. I suggest the claims about repetition as insufficient for learning the goal values be weakened.

Learning over stimulus iterations is possible because participants are told the identity of the goal and nongoal images on each trial so that, in theory, learning can proceed independently of goal repetition. In fact, if goal repetition was necessary for learning, there should not be any performance improvement over stimulus iteration in Experiment 3, where every single trial used new goal/nongoal image pairs. Moreover, in our results from Experiments 5 and 6, where we manipulated the number of times a goal image was presented to participants, we did not find more repetitions to induce better performance.

Nonetheless, repetitions may have a role when the space of concurrently pursued goals is small enough or when repetitions are extremely frequent. In light of this observation, we soften our claims in the Discussion:

> Supplementary Experiments 5 and 6 showed that manipulating the number of times a goal image is experienced does not, by itself, improve learning – in line with our

proposal that reward function compression is necessary for performance gains. *Nonetheless, we note that repetition likely fosters long-term reward functions when goal spaces are small enough.*

In the Results:

Our model predicts that the mere repetition of goals is not *always* sufficient for performance improvements: the goals must be structured in a way that allows for meaningful compression.

And in the Supplementary information:

Contrary to the proposition that simply re-experiencing the same outcome as a goal image strengthens its reinforcing properties, ~~we did not find any effect~~ *any effects of repetitions, if present, were too small to be detected* in Experiment 5.

c) The most compelling evidence for the claim that repetition alone is not enough for goal learning is Experiment 5, which I found the most surprising result in the paper, and which I would suggest deserves a place in the main manuscript rather than the supplementary materials if the authors want to keep this claim. However, I also think this could be better tested by fitting a model to the Experiment 5 data in which separate learning rates are used for trials where the outcomes are the repeated-goal versus novel-goal images within a block. If repeated goals truly confer no benefit for learning, the dual learning rate model should not outperform the baseline with a single learning rate. Otherwise I worry that averaging performance over conditions is obscuring a real repetition effect that the current analyses cannot detect.

We agree with the reviewer that Experiment 5's results are surprising and, in previous iterations, we had drafted a version of the manuscript where all experiments were presented in the main text. However, we found this version distracted the reader from the most important findings. Importantly, while Experiment 5 is a direct test of the repetition effect, we also test (and fail to find) the same effect in Experiment 2, which does appear in the main text. That said, we softened our claims regarding repetition effects as described in response to the previous point.

As for adding analyses within Goal conditions to avoid obscuring repetition effects, we note that these were already present in the manuscript, as highlighted below and in Supplementary Fig. 3. We agree with the reviewer's expectation regarding modeling results. However, because there was no behavioral effect in the direction predicted by the repetitions effect, we find that computational modeling might not only be unnecessary, but risk capturing spurious effects (U-shaped pattern in the relationship between repetitions and performance) that we attribute to noise in the data collection process.

Contrary to the repetitions hypothesis, we found no main effect of stimulus group ($\beta = 0.02 \pm 0.02$, 95% CI = [-0.02, 0.06], $p = 0.355$; Supplementary Fig. 3K-L). *Specifically, although we found a significant difference between the 75%* ($M = 0.66 \pm 0.02$) *and the 50% repetitions groups* ($M = 0.61 \pm 0.02$; $t(50) = 2.27$, $p = 0.027$), *there were no*

significant differences between the 75% and the 25% (M = 0.64 ± 0.02, *t*(50) = 0.97, *p* = 0.335, BF10 = 0.24, *moderate evidence for the null hypothesis*), *or the 50% and 25% repetitions groups* (*t*(50) = 1.68, *p* = 0.1, BF10 = 0.56, *anecdotal evidence for the null hypothesis*).

2. Process of goal learning

While I don't think this is necessary for the study to be published, one limitation of the paper is that the authors offer no model for the process of goal learning itself (by which reward in WM is transferred to memory), through compression or otherwise. I do appreciate the author's usage of reward collection RT as a proxy for compression efficiency, but the mechanism by which goal features are consolidated is left unspecified. One could imagine, for example, a model in which learners incrementally weight stimulus dimensions by their association with Goal, with the effective learning rate for the stimulus-response association on any given trial scaled by the strength of this goal representation. I do not think this model is required for publication, but I do think it would improve the paper to have a discussion of this process, or for the authors to more explicitly acknowledge this as a limitation, particularly given that the current RT models underestimate the condition differences (e.g. Supplementary Fig. 12 and S13), and that the mechanistic claims in the discussion are stated with some confidence.

This point was also raised by Reviewer 1. For ease of review, we copy our response here. To summarize, we agree that this is an important step forward, but we think that it requires developing experiments dedicated to identifying the temporal dynamics of reward collection and its impact on behavior – which the current experiments do not provide. We nonetheless contend that establishing the phenomenon and proposing the conceptual framework are an important step forward.

The reviewer correctly pointed out that we do not establish formal models of compression or long-term memory transfer. While we intend to do so in future work, the current paper focuses on establishing a phenomenon, proposing a conceptual model that can guide follow-up studies, and providing initial evidence in support of the latter. For this reason, we refer to our model as conceptual and explicitly state that algorithmic details are not provided. To make this point even clearer to readers, we have edited our text in several locations (italics for emphasis here only).

In the Abstract, we now say:

> *Although the algorithmic details remain to be established*, our behavioral results and computational models suggest that efficient goal-directed learning relies on compressing complex goal information into a stable reward function.

In the Introduction, we state:

> In our *conceptual* model, a working memory (WM) component enables us to instantly attribute goal-contingent rewarding properties even to abstract, novel

goals. [...] While we remain agnostic with regard to its algorithmic implementation in the brain *and formal instantiations of reward function compression itself*, our model makes specific predictions, which we test in this study.

In Figure 1's caption, we specify:

In our proposed conceptual model, a working memory (WM) component enables us to instantly attribute goal-contingent rewarding properties even to abstract, novel goals.

In the computational modeling section:

Treating reward collection RTs as proxies for reward processing efficiency *(potentially gained through long-term memory transfer)*, we also built a family of models where one of the parameters was directly influenced by the block-wise median (log) reward collection RT for the corresponding condition (i.e., Points, or a specific Goals sub-condition; see Materials and Methods for details) to a degree defined by the parameter ω.

In the Discussion, in three separate paragraphs:

Through a series of experiments and computational modeling, we found ~~strong~~ support for this framework. [...] *However, a formal model of reward functions' long-term memory transfer remains to be established.* [...] Furthermore, the process by which people create compressed reward functions from raw example outcomes – *which we did not model in detail* – remains to be elucidated.

3. Reward collection accuracy as a control

The authors use reward collection d' as a check that goal recognition failures do not explain the learning impairments for arbitrary goals, and argue that the absence of a correlation with learning performance rules this out. I would suggest a more stringent approach on two grounds. First, d' collapses across false positives and false negatives, which may have quite different implications for learning. A participant who frequently collects non-goal outcomes is in a different situation to one who frequently misses goal outcomes, and these should be examined separately within the correlations. Second, beyond the correlation analysis, I would suggest including reward collection accuracy components directly as covariates in the mixed effects models predicting learning performance, for example:

learning accuracy ~ condition + stimulus_iteration + block_number + stimulus_d' + (1|participant)

Where stimulus_d' could be the reward collection accuracy for that particular stimulus within that block, for example. The authors might have better ways of controlling for this, but I think it is important to control for these errors more explicitly within the regression when quantifying the impact of goal condition on learning.

We acknowledge that *d*' is not a perfect measure, and thank the reviewer for suggesting an additional analysis to test the robustness of our findings. In an earlier article using the same data for Experiment 3 here (Molinaro & Collins, *CogSci*, 2023), we offered even stronger and more precise evidence that lapses in goal collection are not the sole culprit for differences in learning performance through computational modeling. Unlike the analysis suggested by the reviewer, this approach uses trial-by-trial (vs. average) measures and avoids collapsing across false positives and false negatives via *d*'. We now mention this result in the present manuscript, but refer the reader to previous work for details:

> In previous work (Molinaro & Collins, *CogSci*, 2023), we also found that a computational model with subjective rewards based on reward collection decisions (i.e., +1 when participants "collected" and +0 when they did not) did not improve performance relative to a baseline model with objective outcome-based feedback.

Minor points

1. A brief definition of d' as the measure of reward collection accuracy (I assume this is z-scored hit rate minus z-scored false alarm rate) would improve accessibility for readers less familiar with signal detection theory.

The first mention of d' in Experiment 3 now reads as:

> d' – the difference between the Z-scored hit and false alarm rates

2. There is a cut-off sentence at the end of the Experiment 5 supplementary text.

The sentence now reads:

> Instead, learning performance was similar across stimulus groups in the Goals condition.

3. The task figure could be improved by adding a clear visual separator to emphasise that the goal/nongoal images are entirely independent of the stimulus-response mapping. This took some time for me to parse and is central to understanding the design. It might also be nice to illustrate the stimulus-response associations themselves i.e. picture-keypress to understand that the fruit/veg are the pictures being associated with keys, and the bottom images are the pictures that form the goal.

To address the reviewer's suggestions, we added a trial to each condition so that the reader can see that goal/nongoal images are independent of the stimulus. We also added key presses showing different correct keys for different stimuli, as well as the go/no-go reward collection response according to the received feedback. To further improve clarity, we explicitly mention these aspects in the caption.

Figure 2: Figure Redacted

4. If I've understood the parameters correctly in Supplementary Fig. 10, the response stickiness parameter η is always negative. This seems like the opposite of what is normally found in RL and I'm wondering if there there is explanation for this. One possibility is that the task structure discourages perseveration for example, if the same picture is never shown twice consecutively and if pictures are always associated with different keys, participants may adopt a habitual switch strategy. If so, it would be helpful to note as it has implications for interpreting the model.

While the above-mentioned figure (now Supplementary Fig. 11) shows that, on average, η is negative, it also illustrates several positive individual data points (blue dots). Moreover, it was possible that the same stimulus would be shown twice consecutively, although this pattern was not very frequent. That said, the reviewer is correct that a deterministic task design might encourage more switching than staying behavior, and that a negative η could capture this prior, as we now acknowledge in the text:

> All free parameters were bounded between 0 and 1, except for η (stickiness parameter), which could take any value between -1 and 1 (*since persistence biases are often found, but our deterministic task design could encourage switch behavior*) and ω, which was constrained between -10 and 10.

Reviewer #2 (Remarks on code availability):

I couldn't see a code base or availability statement linked in the text but there is sufficient detail in the methods.

We thank the reviewer for checking. A zipped folder of the analysis code was submitted with the manuscript. As we now state in the draft, the analysis code (and data) will be shared publicly upon acceptance.

Reviewer #3

This paper investigates whether people learn more slowly when they need to maintain and learn larger sets of goals, compared to situations in which (1) fewer goals are used, (2) goals can be compressed or grouped, or (3) learning can rely on overtrained reward goals such as point systems. The review was written together with a postdoc from the lab. Overall, we found much to like about this work. The studies are well-powered, carefully controlled, and address a fundamental question about the representational constraints underlying learning. Although some of the findings appear quite intuitive, we are not aware of previous studies that systematically mapped out these principles in such a way. As such, the empirical work, together with the modelling, may potentially provide a solid foundation for future work investigating goal-based learning. However, at the same time, we thought the modelling was only briefly motivated and did not really evaluate some of the central theoretical claims that motivated the paper:

Most notably, throughout the manuscript the authors repeatedly emphasize the role of working memory constraints in goal learning and goal compression. In addition, the senior author has previously published highly relevant work on the interaction between reinforcement learning and working memory. For this reason, we were surprised that none of the many models that were compared included an explicit working memory component? While reading the paper, we assumed that one of the core hypotheses under investigation was that larger goal spaces impose greater demands on working memory during reinforcement learning. However, the models compared in the current manuscript do not directly test this hypothesis. Similarly, the idea of goal compression is not really formalized in the modelling. Instead, the modelling did include a Hebbian-learning component, following the recent paper by Collins (2026, Nature Human Behaviour). However, even in that work, the proposed framework still included a working memory component as well, whereas the current model comparisons do not. We therefore believe the paper should either (a) incorporate and directly evaluate a working memory component, or (b) clearly clarify why the current modelling approach does not require this.
Thank you for raising this valid point, which requires clarification. Disentangling WM from RL contributions in learning is only possible in our previous work due to an experimental design where the set size changes across blocks, which impact WM vs. RL differently. Because set sizes were fixed in this study (all experiments used 6 stimuli per block), it was not possible to explicitly tease apart WM and RL contributions computationally. Instead, we

use an RL model with forgetting (φ) as a proxy for the RL + WM mixture. We had partly justified this choice in a footnote, which we now extend and move to the main text.

> We note that this RL component is meant to capture the reward-dependent learning process, with no claim that it maps onto specific underlying neural mechanisms (Eckstein & Collins, 2021; Collins, 2025); indeed, we expect that this RL component additionally reflects contributions of working memory to learning (Collins, 2018). All models also included additional mechanisms, such as forgetting and stickiness, which we found to improve fit. *In particular, forgetting proxies RL-WM mixtures that could not be disentangled due to the fixed set-size design of the experiment.*

Second, we wondered why the model comparison systematically allowed block-specific variation in most model parameters, but not in parameters such as tau or the forgetting rate. Ideally, these parameters should also be allowed to vary across blocks and be included in the comparison procedure.

φ and τ affect learning in a similar way to α and κ. In our preliminary analyses, we found that varying the latter two parameters by condition better captured behavior, so we skipped models with free φ and τ for brevity. We apologize for not justifying this choice explicitly in the initial draft. For completeness, we now include models with free φ and τ. None of our results are affected, as the model with free α still has the lowest AIC (Supplementary Fig. 8).

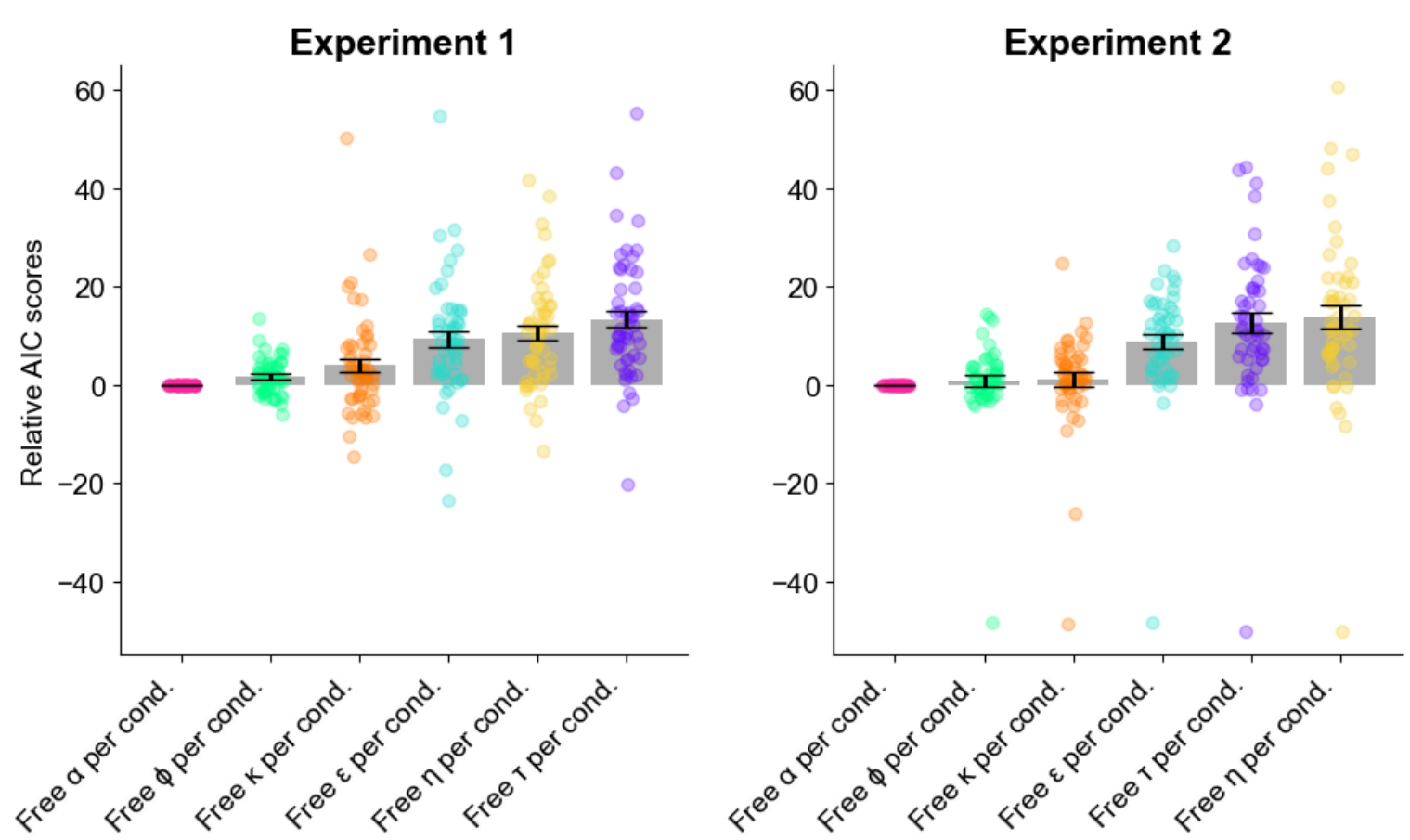

Supplementary Figure 8: Model comparison for Experiments 1 and 2 for models with free parameters for separate Goal sub-conditions. Bars illustrate the average AIC score relative to the best model's across all participants. Dots represent relative AIC scores for individual participants. cond = condition.

Finally, although we believe the central question is fundamentally interesting from a cognitive science perspective, the broader relevance of the work would benefit from the inclusion of a few concrete everyday examples that map closely onto the current paradigm. Providing two or three such examples would help readers connect the experimental manipulations to naturally occurring forms of learning and goal management.

We agree that real-life examples often help readers understand the relevance of a study beyond its contributions to the field. While it is hard to map all features of our task to everyday situations, we did include a real-life example in the Introduction, copied here:

> As a real-life example, consider learning chess: a novice might initially hold specific winning positions in mind ("queen on D5"), then compress these into simple tactical rules ("control the center squares"), and eventually apply such patterns automatically without conscious effort, freeing mental resources for strategic planning.

To avoid cluttering the text with entirely new examples and cause confusion while improving the relatability of the study, we now extend the chess example more specifically in relation to Experiment 1:

> Returning to our chess example, this would be akin to working toward a single target position across many games vs. juggling a repertoire of several distinct target positions. The latter is harder because resources needed for learning must be spared for active goal maintenance.

And Experiment 2:

> Going back to the chess analogy, the two Goals block types are like learning the same number of target positions, but differ in whether all winning positions can be generalized via common features (e.g., controlling the center) or depend on the conjunction of several features, leaving the player to memorize each target board in full.

A very minor point concerns the relation to the action–effect learning literature. We do not believe this literature threatens the novelty of the present work. Nevertheless, some brief discussion of these studies may help situate the manuscript more broadly within existing research on action–outcome representations and learning. The following papers in particular came to mind:

Wolfensteller, U., & Ruge, H. (2011). On the timescale of stimulus-based action–effect learning. Quarterly Journal of Experimental Psychology, 64(7), 1273-1289.

Janczyk, M., Eichfelder, L., Liesefeld, H. R., & Franz, V. H. (2024). Learning and transfer of response–effect relations. Quarterly Journal of Experimental Psychology, 17470218241284259.

Thanks for introducing us to this relevant work. When mentioning the broader literature related to the gradual transfer of reward functions to long-term memory in the Discussion, we now say:

> A similar dynamic occurs in action-effect learning, where outcomes produced by responses gradually become incorporated into action representations within a few trials (Wolfensteller et al., 2011), but only under restricted conditions (Janczyk et al., 2024).

We thank all reviewers for the encouraging words and for acknowledging the value of our work. To address their final suggestions, we report the reviewers' comments verbatim, in black. Our responses are in blue, with new additions from the updated manuscript indented.

Reviewer #1

I appreciate the authors' clear and thoughtful responses to my comments, and the corresponding revisions to the manuscript. While the formal modeling of the goal compression process is deferred to future work — which is a somewhat modest resolution — I recognize that the present study still offers value in establishing the phenomenon and articulating the conceptual framework.

We thank the reviewer for the kind response and for acknowledging the value of our work as it currently stands. We hope that making its main limitation clear from the abstract ("although the algorithmic details remain to be established") helps the reader focus on the insights that our study does present while encouraging follow-up work. We thank the reviewer again for the valuable feedback that helped us improve our manuscript!

Reviewer #2

I thank the authors for their thorough and thoughtful responses, which have addressed nearly all of my concerns. I'm satisfied with the revisions regarding the claims about null repetition effects, the conceptual framing of the models, and all the minor points. I think the task figure is also now very clear.

We thank the reviewer for suggesting useful improvements to our paper and are glad that most points have now been addressed.

I have one remaining request on point 3 i.e. the analyses of incorrect reward collection responses to check the results aren't confounded by confusion about the current goal. While I appreciate the pointer to the trial-by-trial modeling result in Molinaro & Collins (2023), that analysis concerns Experiment 3, whereas my concern is more important for the main experiments, particularly Experiment 2, where reward collection accuracy did differ significantly between conditions. Since the authors already have the relevant trial-level data and mixed-effects models, I think it should be pretty quick to run a control regression like the one I suggested (for example: learning accuracy ~ condition + stimulus iteration + block number + stimulus-level collection accuracy + (1|participant)), or an equivalent specification of the authors' choice e.g. separating the false positives from false negatives within the regression. I expect this will confirm all the existing conclusions, and including it would make the central results feel more robust. With this addition, I'm happy to recommend publication.

We thank the reviewer for clarifying this request and for suggesting a control regression. To address this, we propose a slightly different mixed-effects model with the following formula, tested on a trial-by-trial basis:

`Correct choice ~ Goals condition * stimulus iteration + Goals condition * block number + previous false positive reward collection + previous false negative reward collection + (1 | participant)`

That is, we assess choice correctness based on the main effect of interest (Compressible vs. Incompressible Goals condition) while correcting for whether the *previous* reward collection for the same stimulus resulted in a false positive or a false negative. Our logic is as follows: if the main effects of condition were fully driven by lapses in goal recognition (in turn, leading to poor learning), current accuracy should be explained away by a previous failure in identifying the goal. Note that we include Goals condition interactions with stimulus iteration and block number for consistency with other analyses, but the results reported below hold even without these effects. Compared to stimulus-specific *d*' scores, these indicators address the reviewer's insight that *d*' collapses distinct error types while also avoiding small-cell instability issues of calculating *d*' separately for each stimulus (due to undefined/over-sensitive *d*' with no/few cases of misses and false alarms across the 12 repetitions). This control analysis is particularly conservative: higher WM load in the Incompressible condition, where reward function compression is not possible, might itself produce more collection errors — biasing the analysis against finding a condition effect when controlling for them.

Nevertheless, the results confirm the reviewer's intuition while upholding our primary finding. Reward collection false negatives and false positives had different effects on choice accuracy on the next trial. As expected by the reviewer, the main effect of goal condition (Compressible vs. Incompressible) remained robust, confirming that goal identification lapses do not account for the learning advantages of compressible goal structures. We have updated the manuscript text in the Experiment 2 section as follows:

> Differences in reward collection accuracies based on the type of Goals block were significant ($t(50) = 8.62$, $p < 0.001$; Supplementary Fig. 1). However, they were not significantly correlated with corresponding differences in learning performance ($\rho = 0.17$, $p = 0.226$). *Moreover, a trial-level logistic mixed-effects model predicting choice accuracy confirmed a significant effect of Goals condition (Compressible vs. Incompressible;* $\beta = 0.51 \pm 0.06$, 95% CI =[0.40, 0.63], $p < 0.001$), *even after explicitly controlling for false positive* ($\beta = -0.40 \pm 0.22$, 95% CI = [-0.84, 0.03], $p = 0.071$) *and false negative reward collection errors* ($\beta = -1.58 \pm 0.17$, 95% CI = [-1.91, -1.26], $p < 0.001$) *on the previous encounter of that stimulus (in addition to stimulus iteration, block number, and their interaction with goal condition). These results suggest failures in goal identification are unlikely to fully explain the main effect of Goals condition.*

We hope this addresses the reviewer's final comment, but are available for further clarifications if needed.

Reviewer #3

We would like to thank the authors for engaging with our comments. While we still think a slightly more elaborate modelling approach to support some of the authors' central claims (e.g., about goal compression) would be desirable, we also think the present behavioral findings are interesting and already provide a good foundation for future work investigating goal-based learning.

Similar to our response to Reviewer #1, we thank the reviewer for kindly recognizing our work as a valuable foundation. We made sure to clarify the study's main limitation from the abstract ("although the algorithmic details remain to be established") to help the reader focus on our conceptual framework, behavioral findings, and computational analyses while encouraging follow-up in a currently under-studied domain. We thank the reviewer again for helping us improve our manuscript through their insightful feedback!
